# Effect of 6-Benzoyl-benzothiazol-2-one scaffold on the pharmacological profile of α-alkoxyphenylpropionic acid derived PPAR agonists

**DOI:** 10.1080/14756366.2020.1713771

**Published:** 2020-01-15

**Authors:** Aurélie Hurtevent, Morgan Le Naour, Veronique Leclerc, Pascal Carato, Patricia Melnyk, Nathalie Hennuyer, Bart Staels, Monique Beucher-Gaudin, Daniel-Henri Caignard, Catherine Dacquet, Nicolas Lebegue

**Affiliations:** a Univ. Lille, Inserm, CHU Lille, UMR-S 1172 - JPArc - Centre de Recherche Jean-Pierre AUBERT Neurosciences et Cancer, Lille, France; b Université de Poitiers, CIC INSERM 1402, UFR de Médecine et de Pharmacie, Poitiers, France; c Univ. Lille, Inserm, CHU Lille, Institut Pasteur de Lille, U1011- EGID, Lille, France; d Pôle d’innovation Thérapeutique Maladies Métaboliques, Institut de Recherches Servier, Suresnes, France; e Pôle d'Expertise Chimie Thérapeutique, Institut de Recherches Servier, Croissy-sur seine, France

**Keywords:** Type 2 diabetes, PPAR, SPPARγM, benzothiazol-2-one, body weight gain

## Abstract

A series of nitrogen heterocycles containing α–ethoxyphenylpropionic acid derivatives were designed as dual PPARα/γ agonist ligands for the treatment of type 2 diabetes (T2D) and its complications. 6-Benzoyl-benzothiazol-2-one was the most tolerant of the tested heterocycles in which incorporation of O-methyl oxime ether and trifluoroethoxy group followed by enantiomeric resolution led to the (*S*)-stereoisomer **44 b** displaying the best in vitro pharmacological profile. Compound **44 b** acted as a very potent full PPARγ agonist and a weak partial agonist on the PPARα receptor subtype. Compound **44 b** showed high efficacy in an ob/ob mice model with significant decreases in serum triglyceride, glucose and insulin levels but mostly with limited body-weight gain and could be considered as a selective PPARγ modulator (SPPARγM).

## 1. Introduction

Type 2 diabetes (T2D), is a metabolic disorder characterised by hyperglycaemia caused not only by impaired insulin secretion from the pancreas but also by the increased insulin resistance of peripheral tissues^1^^,^^2^. The peroxisome proliferator-activated receptors (PPARα, PPARβ/δ and PPARγ) are gene-specific transcription factors whose activation by small lipophilic derived ligands leads to significant improvement in insulin resistance, hyperglycaemia^3^, endothelial function^4^, markers of inflammation^5^ and cholesterol metabolism^6^. However, clinical use of PPAR ligands as thiazolidinediones or glitazars has been restricted by their side effect profile, namely, fluid retention, congestive heart failure (CHF), adipogenic weight gain, kidney impairment and a decrease in bone mineral density associated with fractures^7–11^. Recently, INT131 was designed as a selective PPARγ modulator (SPPARγM) to maintain the benefits of the PPARγ full agonists while reducing or deleting their undesirable side effects^12^^,^^13^. This selective PPARγ modulator (SPPARγM) and others^14^ had partial agonist activity and showed increased insulin sensitivity and robust glucose-lowering activity with a reduction of the adverse effects observed with PPARγ full agonists, whatever the animal model of diabetes. These results illustrated that anti-diabetic effects of PPAR ligands can be decoupled from unwanted side effects as oedema or weight gain.

As a part of our programme, we were interested in developing new SPPARγM ligands with full PPARγ agonist activities as well as moderating the adverse side effects as fluid retention or body-weight gain. We have previously described a large series of α–ethoxyphenylpropionic acids bearing a benzoyl-indole core and found that many of them were full dual PPARα/γ agonists with higher selectivity for the PPARγ receptor^15^. Oxime ether introduction and enantiomeric resolution within this series of compounds allowed adjustment of the PPARα/γ potency ratio leading to best equally balanced PPARα/γ ligands. Unfortunately, despite high efficacy in an ob/ob mice model of T2D and dyslipidemia, similar to rosiglitazone and tesaglitazar, these compounds exhibited a disapproving benefit–risk ratio as they displayed a significant increase in body weight gain.

**Figure 1.**
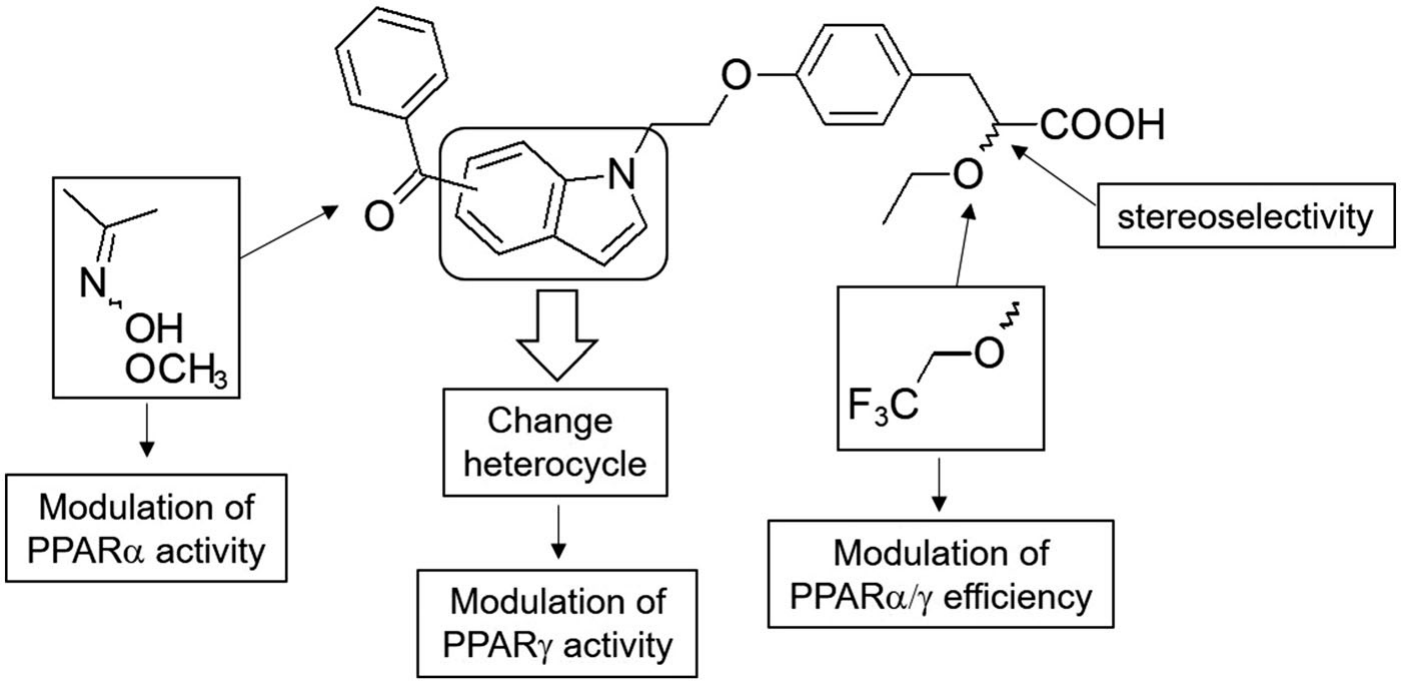
Design of α–alkoxyphenylpropionic acid derivatives bearing various central nitrogen heterocycles.

Herein we investigated the impact of the indole core replacement by various nitrogen heterocycles as benzothiazol-2-one, benzothia- or benzoxazin-3-one and 1,3-dihydro-2*H*-indol-2-one on PPARγ binding affinity and PPARα/γ transactivation assays (Figure 1). The best heterocycle derivative was then selected to further chemical modulations as introduction of oxime and trifluoroethoxy groups as well as synthesis of optically pure enantiomers which were subjected to in vivo studies to assess their anti-hyperlipidemic, anti-hyperglycemic, and body weight effects in experimental animal models.

## 2. Materials and methods

### 2.1. Chemistry

**2.1.1. General**

All reagents and solvents were purchased and used without further purification. Melting points were determined on a BÜCHI B-540 apparatus and are uncorrected. NMR spectra were recorded on a Bruker Avance 300 spectrometer operating at 300 MHz (^1^H) or 75 MHz (^13^C). Chemical shifts are in parts per million (ppm) and were referenced to the residual proton peaks in deuterated solvents. Mass spectra were recorded with an LCMS (Waters Alliance Micromass ZQ 2000). LCMS analysis was performed using a Waters XBridge C18 column (5 µm particle size column, dimensions 50 mm × 4.6 mm). A gradient starting from 98% H_2_O/formate buffer 5 mM (pH 3.8) and reaching 100% CH_3_CN/formate buffer 5 mM (pH 3.8) within 4 min at a flow rate of 2 mL/min was used followed by a return to the starting conditions within 1 min. Elemental analyses were performed by UMR CNRS8181, and were in agreement with the calculated values within ± 0.2%. Synthesis of compounds 1–7 was performed according procedures well described in the literature. Chiral HPLC method: Waters HPLC system was equipped with a HPLC 600 pump and a 2996 photodiode array detector. The HPLC column used was a Daicel Chiralpak AD Column (250 × 4.6 mm). The eluents were: (A) IPA + 0.1% TFA; (B) *n*-hexane. The isocratic condition of elution was at a flow rate of 1 or 1.5 mL/min and at 20 °C with a total run time of 40 min.

**2.1.2. 5-Bromo-3,3-difluoro-1,3-dihydroindol-2-one (9)**

DAST (5.33 mL, 40.7 mmol, 2.3 eq) was added to a solution of 5-bromoisatine (**8**) (4 g, 17.7 mmol, 1.0 eq) in anhydrous DCM (40 mL) and stirred at room temperature for 24 h under inert atmosphere. The reaction mixture was quenched with CH_3_OH (1 mL) then with water (50 mL) and extracted twice with DCM (20 mL). The organic layers were dried over MgSO_4_, filtered and evaporated under reduced pressure. The residue was purified *via* flash column chromatography (cyclohexane/EtOAc 8:2), yielding (**9**) as a yellow solid (3.82 g, 88%); C_8_H_4_BrF_2_NO; MW = 248.03 g/mol; m.p. 195–196 °C. ^1^H NMR (300 MHz, CDCl_3_): *δ* 6.90 (d, 1H, *J* = 8.0 Hz); 7.57 (dd, 1H, *J* = 2.0 Hz, 8.0 Hz); 7.67 (d, 1H, *J* = 2.0 Hz); 8.12 (s, 1H); MS (ESI^+^) *m/z* 247.6 [M + H^79^Br]^+^; 249.8 [M + H^81^Br]^+^.

**2.1.3. 3,3-Difluoro-5-(hydroxyphenylmethyl)-1,3-dihydroindol-2-one (10)**

*n*-Butyllithium (15 mL, 1.6 M in hexane, 24 mmol, 4.0 eq) was added at 0 °C to a solution of *i*-PrMgCl (6 mL, 2 M in THF, 12 mmol, 2.0 eq) and the reaction mixture was stirred for 30 min. A solution of **9** (1.5 g, 6 mmol, 1.0 eq) in anhydrous THF (20 mL) was then added at −10 °C and the reaction mixture was stirred also for 30 min. A solution of benzaldehyde (3.7 mL, 36 mmol, 6.0 eq) in anhydrous THF (20 mL) was added dropwise at −78 °C and the reaction mixture was stirred for 2 h then rised to room temperature. The reaction mixture was quenched with a saturated aqueous ammonium chloride solution (100 mL) and extracted twice with EtOAc (50 mL). The organic layers were dried over MgSO_4_, filtered and evaporated under reduced pressure. The residue was purified *via* flash column chromatography (cyclohexane/EtOAc 6:4), yielding **10** as a yellow solid (1.35 g, 82%); C_15_H_11_F_2_NO_2_; MW= 275.26 g/mol; m.p. 161–163 °C. ^1^H NMR (300 MHz, CDCl_3_): *δ* 5.82 (s, 1H); 6.85 (d, 1H, *J* = 8.2 Hz); 7.30–7.37 (m, 5H); 7.44 (dd, 1H, *J* = 1.6 Hz, 8.2 Hz); 7.57 (d, 1H, *J* = 1.6 Hz); 8.63 (s, 1H); MS (ESI^+^) *m/z* 276.1 [M + H]^+^.

**2.1.4. 5-Benzoyl-3,3-difluoro-1,3-dihydroindol-2-one (11)**

Dess-Martin periodinane (1.1 g, 2.6 mmol, 1.3 eq) dissolved in DCM (20 mL) was added to a solution of **10** (0.55 g, 2.0 mmol, 1.0 eq) and the reaction mixture was stirred at room temperature for 2.5 h. The reaction mixture was quenched with a saturated aqueous ammonium chloride and sodium thiosulphate solution (100 mL) and stirred until a clear solution was obtained. The solution was extracted twice with DCM, the organic layers were washed with a saturated aqueous sodium chloride solution, dried over MgSO_4_, filtered and evaporated under reduced pressure yielding **11** as a yellow solid (0.504 g, 92%) which can be used without further purification. C_15_H_9_F_2_NO_2_; MW= 273.24 g/mol; m.p. 154–156 °C. ^1^H NMR (300 MHz, CDCl_3_): *δ* 7.05 (d, 1H, *J* = 8.2 Hz); 7.52 (m, 2H); 7.63 (m, 1H); 7.75 (m, 2H); 7.98–8.04 (m, 2H); 8.40 (s, 1H); MS (ESI^+^) *m/z* 274.5 [M + H]^+^.

**2.1.5. Ethyl 2-diazo-2-(diethoxyphosphoryl)acetate (12)**

The formation of benzenesulfonyl azide was accomplished according to the published procedure^16^. In brief, benzenesulfonyl chloride (10 g, 56.6 mmol, 1.0 eq) in acetone (100 mL) and sodium azide (5.52 g, 84.9 mmol, 1.5 eq) in water (20 mL) were combined yielding 9.6 g of the desired reagent as a colourless liquid (93%); ^1^H NMR (CDCl_3_): *δ* 7.63 (dd, 2H, *J* = 7.2 and 8.3 Hz), 7.75 (dd, 1H, *J* = 7.2 Hz, *J* = 1.5 Hz), 7.97 (dd, 2H, *J* = 8.3 Hz, *J* = 1.5 Hz). MS *m/z* 184 [M + H]^+^. Next stage was accomplished according to the procedure of Moody *et al.* [48]. Triethylphosphonoacetate (10.4 mL, 52.4 mmol, 1.0 eq) in dry THF (50 mL) was added dropwise to an ice-cold suspension of 60% sodium hydride (3.14 g, 78.6 mmol, 1.5 eq) in dry THF (50 mL). The reaction mixture was stirred at 0 *°*C for 1 h after which time benzenesulfonyl azide (9.6 g, 52.4 mmol, 1.0 eq) in dry THF (100 mL) was added, under a constant flow of N_2_. The reaction mixture was further stirred for 16 h at 0 *°*C then quenched by the addition of 5% aqueous solution of sodium bicarbonate (200 mL). Thereafter, the aqueous phase was extracted with EtOAc which was washed with water and brine. The organic extract was then dried over MgSO_4_, filtered off and concentrated *in vacuo*. The residue was purified *via* flash column chromatography (cyclohexane/EtOAc 5:5), yielding **12** as a pale yellow liquid (11.4 g, 87%); ^1^H NMR (CDCl_3_): *δ* 1.19 (t, 3H, *J* = 7.2 Hz), 1.36 (t, 6H, *J* = 7.2 Hz), 4.18–4.23 (m, 6H). MS *m/z* 251.1 [M + H]^+^.

**2.1.6. General procedure for synthesis of alkoxy phosphonoacetates (13a,b)**

This conversion was performed according to the method of Haigh et al.^17^. A mixture of diazo ester **12** (10 g, 40 mmol), ethanol (23.6 mL, 400 mmol) or 2-trifluoroethanol (28.7 mL, 400 mmol) and Rh(II) acetate dimer (176.8 mg, 0.4 mmol) in toluene (60 mL) was heated with stirring at reflux for 4 h. The solution was concentrated *in vacuo,* hydrolysed and extracted 3 times with EtOAc. The combined organic extracts were washed with water, brine, dried over MgSO_4_, filtered off and concentrated under reduced pressure. The resulting colourless liquid was used in the next step without further purification.

**2.1.7. Ethyl 2-ethoxy-2-(diethylphosphono)acetate (13a)**

Colourless liquid; 80% yield; C_10_H_21_O_6_P; MW= 268.2 g/mol; ^1^H NMR (300 MHz, CDCl_3_): *δ* 1.24–1.35 (m, 12H), 3.55 (m, 1H), 3.70 (m, 1H), 4.23–4.28 (m, 6H), 4.35 (s, 1H). MS (ESI^+^) *m/z* 269.2 [M + H]^+^.

**2.1.8. Ethyl 2,2,2-trifluoroethoxy-2-(diethylphosphono)acetate (13 b)**

Colourless liquid; 80% yield; C_10_H_18_F_3_O_6_P; MW= 322.1 g/mol; ^1^H NMR (300 MHz, CDCl_3_): *δ* 1.24–1.35 (m, 9H), 3.90 (m, 1H), 4.11 (m, 1H), 4.21–4.28 (m, 6H), 4.51 (s, 1H). MS (ESI^+^) *m/z* 323.2 [M + H]^+^.

**2.1.9. 4–(2-Chloroethoxy)benzaldehyde (15)**

A mixture of 4-hydroxybenzaldehyde (14) (10 g, 80 mmol), potassium carbonate (22 g, 160 mmol) and 1-bromo-2-chloroethane (30 mL, 320 mmol) was refluxed in CH_3_CN (80 mL) for 15 h. The reaction mixture was filtered and the filtrate evaporated under reduced pressure. The residue was purified via flash column chromatography (cyclohexane/EtOAc 7:3), yielding **15** as a white powder (13.6 g, 92%); C_9_H_9_ClO_2_; MW= 184.6 g/mol; mp 29–32 °C. ^1^H NMR (300 MHz, CDCl_3_): *δ* 3.86 (t, 2H, *J* = 6.1 Hz), 4.32 (t, 2H, *J* = 6.1 Hz), 7.03 (d, 2H, *J* = 8.9 Hz), 7.86 (d, 2H, *J* = 8.9 Hz), 9.90 (s, 1H). MS (ESI^+^) *m/z* 185 [M + H^35^Cl]^+^, 187 [M + H^37^Cl]^+^.

**2.1.10. General procedure for synthesis of propionate (17a,b)**

Phosphonoacetate **13a** (5 g, 18.6 mmol) or **13 b** (6 g, 18.6 mmol) in THF (50 mL) was added dropwise to a suspension of 60% sodium hydride (0.93 g, 23.2 mmol, 1.5 eq) in anhydrous THF (100 mL) at 0 *°*C under a constant flow of N_2_ and the reaction mixture was stirred at 0 *°*C for 1 h. Benzaldehyde **15** (2.85 g, 15.5 mmol) was then added in dry THF (50 mL). Post stirring for a further 4 h at room temperature, the solution was concentrated *in vacuo* and the residue dissolved in EtOAc for washing with water and brine. Combined organic extracts were dried over MgSO_4_, filtered off and concentrated *in vacuo* leading a residue that was purified by flash column (cyclohexane/EtOAc 8:2) to yield 16a,b (66–92% yield) as mixtures of two isomers (ratio *Z: E* = 60: 40) which were not separated and directly used in the next catalytic hydrogenation step. A solution of **16a,b** (13.2 mmol) in ethanol (150 mL) was stirred with 10% Pd/C catalyst at room temperature under an atmospheric pressure of hydrogen for 20 h. The catalyst is separated by filtration and the solvent evaporated under reduced pressure. The crude product was purified *via* flash column chromatography (cyclohexane/EtOAc 7:3).

**2.1.11. Ethyl 3-[4–(2-chloroethoxy)-phenyl]-2-ethoxy-propionate (17a)**

Colourless liquid; 93% yield; C_15_H_21_ClO_4_; MW= 300.8 g/mol; ^1^H NMR (300 MHz, CDCl_3_): *δ* 1.17 (t, 3H, *J* = 7.1 Hz), 1.24 (t, 3H, *J* = 7.1 Hz), 2.97 (d, 2H, *J* = 5.6 Hz), 3.33 (m, 1H), 3.59 (m, 1H), 3.81 (t, 2H, *J* = 5.9 Hz), 3.98 (t, 1H, *J* = 5.6 Hz), 4.12–4.25 (m, 4H), 6.85 (d, 2H, *J* = 8.6 Hz), 7.17 (d, 2H, *J* = 8.6 Hz); MS (ESI^+^) *m/z* 300.1 [M + H^35^Cl]^+^, 302.2 [M + H^37^Cl]^+^.

**2.1.12. Ethyl 3-[4–(2-chloroethoxy)-phenyl]-2,2,2-trifluoroethoxy-propionate (17 b)**

Colourless liquid; 97% yield; C_15_H_18_ClF_3_O_4_; MW= 354.8 g/mol; ^1^H NMR (300 MHz, CDCl_3_): *δ* 1.25 (t, 3H, *J* = 7.1 Hz), 3.02 (m, 2H), 3.69 (m, 1H), 3.80 (t, 2H, *J* = 5.9 Hz), 3.99 (m, 1H), 4.18 (m, 5H), 6.84 (d, 2H, *J* = 8.8 Hz), 7.16 (d, 2H, *J* = 8.8 Hz); MS (ESI^+^) *m/z* 354.2 [M + H^35^Cl]^+^, 356.2 [M + H^37^Cl]^+^.

**2.1.13. General procedure for synthesis of propionic acid (18a,b)**

Lithium hydroxide (0.49 g, 20.4 mmol, 3.0 eq) was added to a solution of **17a** (2 g, 6.6 mmol, 1.0 eq) or **17 b** (2.36 g, 6.6 mmol, 1.0 eq) dissolved in a 1: 1 mixture of THF (30 mL) and water (30 mL), and the mixture was then stirred at room temperature for 20 h. Thereafter the whole was concentrated *in vacuo*, and the concentrate subjected to vigorous stirring at 0 °C as the solution pH was adjusted to 1 with additions of aliquots of 1 M HCl. The resulting precipitate was filtered, washed with water, pentane and dried under reduced pressure.

**2.1.14. 3-[4–(2-Chloroethoxy)-phenyl]-2-ethoxy-propionic acid (18a)**

White solid; 85%; C_13_H_17_ClO_4_; MW = 272.72 g/mol; mp 49–51 °C; ^1^H NMR (DMSO-*d*_6_): *δ* 1.04 (t, 3H, *J* = 7.0 Hz), 2.83 (m, 2H), 3.29 (m, 1H), 3.52 (m, 1H), 3.92 (t, 2H, *J* = 5.5 Hz), 4.03 (m, 1H), 4.21 (t, 2H, *J* = 5.5 Hz), 6.87 (d, 2H, *J* = 8.5 Hz), 7.14 (d, 2H, *J* = 8.5 Hz); MS (ESI^+^) *m/z* 273.2 [M + H^35^Cl]^+^, 275.3 [M + H^37^Cl]^+^.

**2.1.15. 3-[4–(2-Chloroethoxy)-phenyl]-2,2,2-trifluoroethoxy-propionic acid (18 b)**

White solid; 91%; C_13_H_14_ClF_3_O_4_; MW= 326.72 g/mol; mp 70–72 °C; ^1^H NMR (CDCl_3_): *δ* 3.03 (dd, 1H, *J* = 8.05 Hz, 14.4 Hz), 3.16 (dd, 1H, *J* = 4 Hz, 14.4 Hz); 3.71 (m, 1H), 3.81 (t, 2H, *J* = 5.85 Hz), 4.01 (m, 1H), 4.22 (m, 3H), 6.87 (d, 2H, *J* = 8.7 Hz), 7.19 (d, 2H, *J* = 8.7 Hz), 9.85 (s, 1H); MS (ESI^+^) *m/z* 327.3 [M + H^35^Cl]^+^, 329.4 [M + H^37^Cl]^+^.

**2.1.16. General procedure for synthesis of diastereoisomers (19a,b and 20a,b)**

To a solution of **18a** (3.4 g, 12.5 mmol, 1.0 eq) or **18 b** (4.09 g, 12.5 mmol, 1.0 eq) in DCM (150 mL) at 0 °C; EDCI (2.87 g, 15.0 mmol, 1.2 eq), (*S*)-(-)-2-phenylgycinol (1.74 g, 12.5 mmol, 1.0 eq), and HOBT (2.0 g, 15.0 mmol, 1.2 eq) were added. Finally, triethylamine (2.1 mL, 15.0 mmol, 1.2 eq) was added dropwise at 0 °C. The reaction mixture was then stirred for 12 h at ambient temperature, quenched with saturated aqueous ammonium chloride solution, and diluted with DCM. The organic layer was washed with water, dried over MgSO_4_, filtered, concentrated *in vacuo* and the resulting residue was purified *via* flash column chromatography (petroleum ether/EtOAc 45:55).

**2.1.17. 2-(S)-3-[4–(2-Chloroethoxy)-phenyl]-2-ethoxy-N-((S)-2-hydroxy-1-phenylethyl) propanamide (19a)**

Off-white solid; 37%; C_21_H_26_ClNO_4_; MW= 391.88 g/mol; mp 135–137 °C; ^1^H NMR (CDCl_3_): *δ* 1.21 (t, 3H, *J* = 7.0 Hz), 2.51 (m, 1H); 2.91 (dd, 1H, *J* = 6.75 Hz, 14.3 Hz), 3.09 (dd, 1H, *J* = 3.75 Hz, 14.3 Hz), 3.58 (m, 2H), 3.85 (m, 4H), 4.02 (dd, 1H, *J* = 3.75 Hz, 6.75 Hz), 4.19 (t, 2H, *J* = 5.8 Hz), 5.01 (m, 1H), 6.78 (d, 2H, *J* = 8.7 Hz), 7.07 (dd, 2H, *J* = 2.0 Hz, 7.6 Hz), 7.13 (m, 3H), 7.33 (m, 3H); MS (ESI^+^) *m/z* 392.3 [M + H^35^Cl]^+^, 394.3 [M + H^37^Cl]^+^.

**2.1.18. 2-(S)-3-[4–(2-Chloroethoxy)-phenyl]-2,2,2-trifluoroethoxy-N-((S)-2-hydroxy-1-phenylethyl) propanamide (19 b)**

Off-white solid; 37%; C_21_H_23_ClF_3_NO_4_; MW= 445.86 g/mol; mp 139–140 °C; ^1^H NMR (CDCl_3_): *δ* 2.21 (t, 1H, *J* = 6.85 Hz), 2.94 (dd, 1H, *J* = 7.25 Hz, 14.55 Hz), 3.16 (dd, 1H, *J* = 3.45 Hz, 14.55 Hz), 3.75–3.88 (m, 6H), 4.14–4.22 (m, 3H), 5.03 (m, 1H), 6.79 (d, 2H, *J* = 8.55 Hz), 7.00 (d, 1H, *J* = 7.25 Hz), 7.07–7.13 (m, 4H), 7.33 (m, 3H); MS (ESI^+^) *m/z* 447.1 [M + H^35^Cl]^+^, 449.0 [M + H^37^Cl]^+^.

**2.1.19. 2-(R)-3-[4–(2-Chloroethoxy)-phenyl]-2-ethoxy-N-((S)-2-hydroxy-1-phenylethyl) propanamide (20a)**

Off-white solid; 33%; C_21_H_26_ClNO_4_; MW = 391.88 g/mol; mp 127–128 °C; ^1^H NMR (CDCl_3_): *δ* 1.16 (t, 3H, *J* = 7.0 Hz), 2.48 (dd, 1H, *J* = 5.25 Hz, 7.5 Hz); 2.96 (dd, 1H, *J* = 6.4 Hz, 14.3 Hz), 3.15 (dd, 1H, *J* = 4.0 Hz, 14.3 Hz), 3.52 (m, 2H), 3.71 (m, 2H), 3.83 (t, 2H, *J* = 5.8 Hz), 4.0 (dd, 1H, *J* = 4.0 Hz, 6.4 Hz), 4.24 (t, 2H, *J* = 5.8 Hz), 4.99 (m, 1H), 6.88 (d, 2H, *J* = 8.7 Hz), 7.03 (d, 2H, *J* = 7.3 Hz), 7.20 (m, 3H), 7.34 (m, 3H); MS (ESI^+^) *m/z* 392.3 [M + H^35^Cl]^+^, 394.3 [M + H^37^Cl]^+^.

**2.1.20. 2-(R)-3-[4–(2-Chloroethoxy)-phenyl]-2,2,2-trifluoroethoxy-N-((S)-2-hydroxy-1-phenylethyl) propanamide (20 b)**

Off-white solid; 37%; C_21_H_23_ClF_3_NO_4_; MW= 445.86 g/mol; mp 126–127 °C; ^1^H NMR (CDCl_3_): *δ* 2.11 (t, 1H, *J* = 6.35 Hz), 3.02 (dd, 1H, *J* = 6.75 Hz, 14.2 Hz), 3.21 (dd, 1H, *J* = 3.7 Hz, 14.2 Hz), 3.66–3.75 (m, 3H), 3.78–3.85 (m, 3H), 4.13 (dd, 1H, *J* = 3.7 Hz, 6.35 Hz), 4.23 (t, 2H, *J* = 5.9 Hz), 5.01 (m, 1H), 6.89 (d, 2H, *J* = 8.8 Hz), 6.93 (d, 1H, *J* = 7.35 Hz), 7.18–7.22 (m, 4H), 7.29–7.37 (m, 3H); MS (ESI^+^) *m/z* 447.1 [M + H^35^Cl]^+^, 449.0 [M + H^37^Cl]^+^.

**2.1.21. General procedure for synthesis of optically pure propionic acids (21a,b and 22a,b)**

5M aqueous solution of H_2_SO_4_ (82 mL, 408 mmol, 100 eq) was added to a solution of **19a** or **20a** (1.6 g, 4.08 mmol, 1 eq) or 19 b or 20 b (1.8 g, 4.08 mmol, 1 eq) in a 1: 1 mixture of 1,4-dioxane (80 mL) and water (80 mL). The reaction mixture was refluxed for 8 h, cooled to room temperature, diluted with EtOAc, and the resulting organic layer was washed with H_2_O, dried over MgSO_4_, filtered, concentrated *in vacuo* and the brown residue was purified by flash column chromatography (DCM/MeOH/NEt_3_ 96:2:2).

**2.1.22. 2-(S)- 3-[4–(2-Chloroethoxy)-phenyl]-2-ethoxypropionic acid (21a)**

White solid; 81%; C_13_H_17_ClO_4_; MW= 272.72 g/mol; mp 49–51 °C; ^1^H NMR (DMSO-*d*_6_): *δ* 1.04 (t, 3H, *J* = 7.0 Hz), 2.83 (m, 2H), 3.29 (m, 1H), 3.52 (m, 1H), 3.92 (t, 2H, *J* = 5.5 Hz), 4.03 (m, 1H), 4.21 (t, 2H, *J* = 5.5 Hz), 6.87 (d, 2H, *J* = 8.5 Hz), 7.14 (d, 2H, *J* = 8.5 Hz); MS (ESI^+^) *m/z* 273.2 [M + H^35^Cl]^+^, 275.3 [M + H^37^Cl]^+^. Chiral HPLC method: flow rate= 1.0 mL/min, eluents = A/B 5:95, *t*_r_= 16.62 min.

**2.1.23. 2-(S)- 3-[4–(2-Chloroethoxy)-phenyl]- 2,2,2-trifluoroethoxypropionic acid (21b)**

White solid; 84%; C_13_H_14_ClF_3_O_4_; MW= 326.72 g/mol; mp 70–72 °C; ^1^H NMR (CDCl_3_): *δ* 3.03 (dd, 1H, *J* = 8.05 Hz, 14.4 Hz), 3.16 (dd, 1H, *J* = 4 Hz, 14.4 Hz); 3.71 (m, 1H), 3.81 (t, 2H, *J* = 5.85 Hz), 4.01 (m, 1H), 4.22 (m, 3H), 6.87 (d, 2H, *J* = 8.7 Hz), 7.19 (d, 2H, *J* = 8.7 Hz), 9.85 (s, 1H); MS (ESI^+^) *m/z* 327.3 [M + H^35^Cl]^+^, 329.4 [M + H^37^Cl]^+^. Chiral HPLC method: flow rate= 1.0 mL/min, eluents = A/B 5:95, *t*_r_= 14.85 min.

**2.1.24. 2-(R)- 3-[4–(2-Chloroethoxy)-phenyl]-2-ethoxypropionic acid (22a)**

White solid; 78%; C_13_H_17_ClO_4_; MW = 272.72 g/mol; mp 49–51 °C; ^1^H NMR (DMSO-*d*_6_): *δ* 1.04 (t, 3H, *J* = 7.0 Hz), 2.83 (m, 2H), 3.29 (m, 1H), 3.52 (m, 1H), 3.92 (t, 2H, *J* = 5.5 Hz), 4.03 (m, 1H), 4.21 (t, 2H, *J* = 5.5 Hz), 6.87 (d, 2H, *J* = 8.5 Hz), 7.14 (d, 2H, *J* = 8.5 Hz); MS (ESI^+^) *m/z* 273.2 [M + H^35^Cl]^+^, 275.3 [M + H^37^Cl]^+^. Chiral HPLC method: flow rate= 1.0 mL/min, eluents = A/B 5:95, *t*_r_= 12.48 min.

**2.1.25. 2-(R)- 3-[4–(2-Chloroethoxy)-phenyl]- 2,2,2-trifluoroethoxypropionic acid (22 b)**

White solid; 68%; C_13_H_14_ClF_3_O_4_; MW = 326.72 g/mol; mp 70–72 °C; ^1^H NMR (CDCl_3_): *δ* 3.03 (dd, 1H, *J* = 8.05 Hz, 14.4 Hz), 3.16 (dd, 1H, *J* = 4 Hz, 14.4 Hz); 3.71 (m, 1H), 3.81 (t, 2H, *J* = 5.85 Hz), 4.01 (m, 1H), 4.22 (m, 3H), 6.87 (d, 2H, *J* = 8.7 Hz), 7.19 (d, 2H, *J* = 8.7 Hz), 9.85 (s, 1H); MS (ESI^+^) *m/z* 327.3 [M + H^35^Cl]^+^, 329.4 [M + H^37^Cl]^+^. Chiral HPLC method: flow rate= 1.0 mL/min, eluents = A/B 5:95, *t*_r_= 12.63 min.

**2.1.26. General procedure for synthesis of N-alkylated benzoyl heterocycles (23–30)**

To a solution of benzoyl heterocycle derivative **1–7** or **11** (0.5 g, 2.17 mmol, 1.0 eq) in DMF (35 mL) was added potassium carbonate (0.9 g, 6.5 mmol, 3 eq) and the mixture was stirred for 1 h at 100 °C. A solution of 17a (0.77 g, 2.6 mmol, 1.2 eq) or 17 b (0.85 g, 2.6 mmol, 1.2 eq) in DMF (5 mL) was added dropwise and the mixture was stirred at 120 °C for 15 h. After cooling, the reaction mixture was filtered, hydrolysed and extracted twice with EtOAc. The combined organic extracts were washed with water, dried over MgSO_4_, filtered off and concentrated *in vacuo*. The crude product was purified *via* flash column chromatography (cyclohexane/EtOAc 7:3) to afford the corresponding *N*-alkylated heterocycle derivatives.

**2.1.27. Ethyl 3-{4-[2–(5-benzoyl-2-oxo-benzothiazol-3-yl)-ethoxy]-phenyl}-2-ethoxypropionate (23)**

Colourless gum; 58% yield; C_29_H_29_NO_6_S; MW= 519.62 g/mol; ^1^H NMR (CDCl_3_): *δ* 1.16 (t, 3H, *J* = 7 Hz); 1.24 (t, 3H, *J* = 7 Hz); 2.93 (d, 2H, *J* = 6.1 Hz); 3.35 (m, 1H); 3.59 (m, 1H); 3.95 (t, 1H, *J* = 6.1 Hz); 4.16 (q, 2H, *J* = 7 Hz); 4.29 (t, 2H, *J* = 5 Hz); 4.42 (t, 2H, *J* = 5 Hz); 6.74 (d, 2H, *J* = 8.5 Hz); 7.12–7.16 (m, 4H); 7.25 (d, 1H, *J* = 7.9 Hz); 7.52 (d, 2H, *J* = 7.6 Hz); 7.60 (dd, 1H, *J* = 1.5 Hz, 7.9 Hz); 7.83 (dd, 1H, *J* = 1.2 Hz, 8.2 Hz); 7.90 (d, 1H, *J* = 1.5 Hz); MS (ESI^+^) *m/z* 520.3 [M + H]^+^.

**2.1.28. Ethyl 3-{4-[2–(6-benzoyl-2-oxo-benzothiazol-3-yl)-ethoxy]-phenyl}-2-ethoxypropionate (24a)**

Colourless gum; 61% yield; C_29_H_29_NO_6_S; MW= 519.62 g/mol; ^1^H NMR (CDCl_3_): *δ* 1.15 (t, 3H, *J* = 7.05 Hz); 1.24 (t, 3H, *J* = 7.05 Hz); 2.93 (d, 2H, *J* = 6.15 Hz); 3.33 (m, 1H); 3.59 (m, 1H); 3.94 (t, 1H, *J* = 6.5 Hz); 4.16 (q, 2H, *J* = 7.05 Hz); 4.30 (t, 2H, *J* = 5.05 Hz); 4.40 (t, 2H, *J* = 5.05 Hz); 6.74 (d, 2H, *J* = 9 Hz); 7.14 (d, 2H, *J* = 9 Hz); 7.42 (d, 1H, *J* = 8.45 Hz); 7.51 (t, 2H, *J* = 8 Hz); 7.62 (t, 1H, *J* = 7.5 Hz); 7.79 (d, 2H, *J* = 8 Hz); 7.87 (dd, 1H, *J* = 1.65 Hz, 8.45 Hz); 7.93 (d, 1H, *J* = 1.65 Hz). MS (ESI^+^) *m/z* 520.3 [M + H]^+^.

**2.1.29. Ethyl 3-{4-[2–(6-benzoyl-2-oxo-benzothiazol-3-yl)-ethoxy]-phenyl}-2–(2,2,2-trifluoroethoxy)propionate (24 b)**

Colourless gum; 64% yield; C_29_H_26_F_3_NO_6_S; MW= 573.58 g/mol; ^1^H NMR (CDCl_3_): *δ* 1.25 (t, 3H, *J* = 7.1 Hz); 3.01 (m, 2H); 3.67 (m, 1H); 4.00 (m, 1H), 4.11–4.22 (m, 3H), 4.30 (t, 2H, *J* = 4.55 Hz); 4.40 (t, 2H, *J* = 4.55 Hz); 6.75 (d, 2H, *J* = 8.8 Hz); 7.13 (d, 2H, *J* = 8.8 Hz); 7.41 (d, 1H, *J* = 8.55 Hz); 7.51 (t, 2H, *J* = 7.6 Hz); 7.62 (t, 1H, *J* = 7.6 Hz); 7.79 (d, 2H, *J* = 8.2 Hz); 7.87 (dd, 1H, *J* = 1.75 Hz, 8.55 Hz); 7.93 (d, 1H, *J* = 1.75 Hz). MS (ESI^+^) *m/z* 574.3 [M + H]^+^.

**2.1.30. Ethyl 3-{4-[2–(6-benzoyl-3-oxo-benzo[1, 4]thiazin-4-yl)-ethoxy]-phenyl}-2-ethoxypropionate (25)**

Colourless gum; 48% yield; C_30_H_31_NO_6_S; MW= 533.65 g/mol; ^1^H NMR (CDCl_3_): *δ* 1.16 (t, 3H, *J* = 7 Hz); 1.25 (t, 3H, *J* = 7.2 Hz); 2.93 (d, 2H, *J* = 6.2 Hz); 3.33 (m, 1H); 3.48 (s, 2H); 3.61 (m, 1H); 3.95 (t, 1H, *J* = 6.2 Hz); 4.18 (q, 2H, *J* = 7.2 Hz); 4.29 (t, 2H, *J* = 4.8 Hz); 4.36 (t, 2H, *J* = 4.8 Hz); 6.68 (d, 2H, *J* = 8.3 Hz); 7.11 (d, 2H, *J* = 8.3 Hz); 7.19 (m, 2H); 7.51 (dd, 2H, *J* = 7.5 Hz, 8.3 Hz); 7.62 (dd, 1H, *J* = 1.5 Hz, 7.5 Hz); 7.84 (dd, 2H, *J* = 1.5 Hz, 8.3 Hz); 8.25 (d, 1H, *J* = 1.8 Hz). MS (ESI^+^) *m/z* 534.3 [M + H]^+^.

**2.1.31. Ethyl 3-{4-[2–(7-benzoyl-3-oxo-benzo[1, 4]thiazin-4-yl)-ethoxy]-phenyl}-2-ethoxypropionate (26)**

Colourless gum; 78% yield; C_30_H_31_NO_6_S; MW= 533.65 g/mol; ^1^H NMR (CDCl_3_): *δ* 1.16 (t, 3H, *J* = 7.1 Hz); 1.24 (t, 3H, *J* = 7.1 Hz); 2.95 (d, 2H, *J* = 5.7 Hz); 3.34 (m, 1H); 3.46 (s, 2H); 3.61 (m, 1H); 3.96 (t, 1H, *J* = 5.7 Hz); 4.17 (q, 2H, *J* = 7.1 Hz); 4.32 (t, 2H, *J* = 4.6 Hz); 4.39 (t, 2H, *J* = 4.6 Hz); 6.81 (d, 2H, *J* = 8.6 Hz); 7.16 (d, 2H, *J* = 8.6 Hz); 7.50 (m, 2H); 7.63 (dd, 1H, *J* = 1.4 Hz, 7.1 Hz); 7.69 (d, 1H, *J* = 8.7 Hz); 7.76 (dd, 1H, *J* = 1.7 Hz, 8.7 Hz); 7.80 (dd, 2H, *J* = 1.3 Hz, 7 Hz); 7.86 (d, 1H, *J* = 1.7 Hz). MS (ESI^+^) *m/z* 534.3 [M + H]^+^.

**2.1.32. Ethyl 3-{4-[2–(6-benzoyl-3-oxo-benzo[1, 4]oxazin-4-yl)-ethoxy]-phenyl}-2-ethoxypropionate (27)**

Colourless gum; 73% yield; C_30_H_31_NO_7_; MW= 517.58 g/mol; ^1^H NMR (CDCl_3_): *δ* 1.16 (t, 3H, *J* = 7.1 Hz); 1.24 (t, 3H, *J* = 7.2 Hz); 2.95 (d, 2H, *J* = 5.3 Hz); 3.34 (m, 1H); 3.60 (m, 1H); 3.95 (t, 1H, *J* = 5.3 Hz); 4.15 (q, 2H, *J* = 5.2 Hz); 4.26 (t, 2H, *J* = 5.2 Hz); 4.37 (t, 2H, *J* = 5.2 Hz); 4.72 (s, 2H); 6.75 (d, 2H, *J* = 9.1 Hz); 7.06 (d, 1H, *J* = 8.1 Hz); 7.13 (d, 2H, *J* = 9.1 Hz); 7.27 (dd, 1H, *J* = 1.9 Hz, 8.1 Hz); 7.48 (m, 2H); 7.60 (m, 1H); 7.81 (m, 2H); 8.03 (d, 1H, *J* = 1.9 Hz). MS (ESI^+^) *m/z* 518.4 [M + H]^+^.

**2.1.33. Ethyl 3-{4-[2–(7-benzoyl-3-oxo-benzo[1, 4]oxazin-4-yl)-ethoxy]-phenyl}-2-ethoxypropionate (28)**

Colourless gum; 69% yield; C_30_H_31_NO_7_; MW = 517.58 g/mol; ^1^H NMR (CDCl_3_): *δ* 1.16 (t, 3H, *J* = 6.85 Hz); 1.24 (t, 3H, *J* = 7.2 Hz); 2.94 (m, 2H); 3.34 (m, 1H); 3.60 (m, 1H); 3.95 (dd, 1H, *J* = 6 Hz, 7.3 Hz); 4.17 (q, 2H, *J* = 7.2 Hz); 4.28 (t, 2H, *J* = 5.05 Hz); 4.37 (t, 2H, *J* = 5.05 Hz); 4.68 (s, 2H); 6.79 (d, 2H, *J* = 8.7 Hz); 7.15 (d, 2H, *J* = 8.7 Hz); 7.45 (d, 1H, *J* = 8.4 Hz); 7.47–7.53 (m, 3H); 7.57 (dd, 1H, *J* = 1.9 Hz, 8.4 Hz); 7.61 (t, 1H, *J* = 7.2 Hz); 7.79 (dd, 2H, *J* = 1.45 Hz, 8.4 Hz). MS (ESI^+^) *m/z* 518.4 [M + H]^+^.

**2.1.34. Ethyl 3-{4-[2–(5-benzoyl-2-oxo-2,3-dihydroindol-1-yl)ethoxy]phenyl}-2-ethoxypropionate (29)**

Colourless gum; 68% yield; C_30_H_31_NO_6_; MW= 501.57 g/mol; 1H NMR (CDCl_3_): *δ* 1.15 (t, 3H, *J* = 7 Hz); 1.25 (t, 3H, *J* = 7.3 Hz); 2.64 (s, 2H); 2.93 (m, 2H); 3.44 (m, 1H); 3.59 (m, 1H); 3.94 (m, 1H); 4.12–4.23 (m, 4H); 4.28 (t, 2H, *J* = 7 Hz); 6.73 (d, 2H, *J* = 8.8 Hz); 7.14 (d, 2H, *J* = 8.8 Hz); 7.30 (d, 1H, *J* = 8.5 Hz); 7.52 (m, 2H); 7.60 (m, 1H); 7.78 (m, 2H); 8.01 (d, 1H, *J* = 1.4 Hz); 8.28 (dd, 1H, *J* = 1.4 Hz, 8.5 Hz). MS (ESI^+^) *m/z* 502.4 [M + H]^+^.

**2.1.35. Ethyl 3-{4-[2–(5-benzoyl-3,3-difluoro-2-oxo-2,3-dihydroindol-1-yl)ethoxy]phenyl}-2-ethoxypropionate (30)**

Colourless gum; 62% yield; C_30_H_29_F_2_NO_6_; MW= 537.57 g/mol; 1H NMR (CDCl_3_): *δ* 1.15 (t, 3H, *J* = 7 Hz); 1.25 (t, 3H, *J* = 7.3 Hz); 2.93 (m, 2H); 3.32 (m, 1H); 3.59 (m, 1H); 3.94 (m, 1H); 4.12–4.23 (m, 4H); 4.28 (t, 2H, *J* = 7 Hz); 6.73 (d, 2H, *J* = 8.8 Hz); 7.14 (d, 2H, *J* = 8.8 Hz); 7.30 (d, 1H, *J* = 8.5 Hz); 7.52 (m, 2H); 7.60 (m, 1H); 7.78 (m, 2H); 8.01 (d, 1H, *J* = 1.4 Hz); 8.08 (dd, 1H, *J* = 1.4 Hz, 8.5 Hz). MS (ESI^+^) *m/z* 538.3 [M + H]^+^.

**2.1.36. General procedure for synthesis of carboxylic acids (31–38)**

A solution of ester **23–30** (0.5 mmol, 1.0 eq) and lithium hydroxide (36 mg, 1.5 mmol, 3.0 eq) in THF/H_2_O (3:2, 15 mL) is stirred at room temperature for 10 h. After removal of the solvent *in vacuo*, 1 N HCl aqueous solution (10 mL) is added and the resulting precipitate is filtered and purified by flash column chromatography (DCM/MeOH 9:1) to yield the corresponding carboxylic acid.

**2.1.37. 3-{4-[2–(5-benzoyl-2-oxo-benzothiazol-3-yl)-ethoxy]-phenyl}-2-ethoxypropionic acid (31)**

Pale yellow solid; 82% yield; C_27_H_25_NO_6_S; MW = 491.57 g/mol; m.p. 49–51 °C. ^1^H NMR (CDCl_3_): *δ* 1.18 (t, 3H, *J* = 7 Hz); 3.06 (d, 2H, *J* = 6.4 Hz); 3.53 (m, 2H); 4.11 (t, 1H, *J* = 6.4 Hz); 4.31 (t, 2H, *J* = 5 Hz); 4.42 (t, 2H, *J* = 5 Hz); 6.76 (d, 2H, *J* = 8.8 Hz); 7.10–7.13 (m, 4H); 7.23–7.26 (m, 4H); 7.83 (dd, 1H, *J* = 1.5 Hz, 7 Hz); 7.88 (d, 1H, *J* = 1.5 Hz); ^13 ^C NMR (75 MHz, CDCl_3_): *δ* 15.0, 37.6, 42.8, 65.5, 66.9, 79.6, 112.6, 114.3, 121.9, 125.4, 128.4, 129.9, 130.0, 130.6, 132.6, 134.6, 135.8, 137.4, 137.9, 156.9, 169.8, 173.7, 195.7. *t*_R,LCMS_ = 2.44 min; MS (ESI^+^) *m/z* 492.3 [M + H]^+^. Anal. Calcd (%) for C_27_H_25_NO_6_S: C, 65.97; H, 5.13; N, 2.85; found C, 65.63; H, 5.31; N, 2.64.

**2.1.38. 3-{4-[2–(6-benzoyl-2-oxo-benzothiazol-3-yl)-ethoxy]-phenyl}-2-ethoxypropionic acid (32a)**

Off-white solid; 79% yield; C_27_H_25_NO_6_S; MW= 491.57 g/mol; m.p. 57–59 °C. ^1^H NMR (CDCl_3_): *δ* 1.16 (t, 3H, *J* = 6.95 Hz); 2.93 (dd, 1H, *J* = 7.55 Hz, 14.05 Hz); 3.04 (dd, 1H, *J* = 4.4 Hz, 14.05 Hz); 3.41 (m, 1H); 3.60 (m, 1H); 4.02 (dd, 1H, *J* = 4.4 Hz, 7.65 Hz); 4.30 (t, 2H, *J* = 5.45 Hz); 4.40 (t, 2H, *J* = 5.45 Hz); 6.74 (d, 2H, *J* = 8.5 Hz); 7.13 (d, 2H, *J* = 8.5 Hz); 7.41 (d, 1H, *J* = 8.6 Hz); 7.51 (t, 2H, *J* = 7.8 Hz); 7.62 (t, 1H, *J* = 7.5 Hz); 7.78 (d, 2H, *J* = 8 Hz); 7.86 (dd, 1H, *J* = 1.8 Hz, 8.5 Hz); 7.92 (d, 1H, *J* = 1.8 Hz). ^13 ^C NMR (75 MHz, CDCl_3_): *δ* 15.0, 37.7, 42.9, 65.5, 66.7, 79.6, 111.1, 114.2, 122.6, 124.8, 128.4, 129.0, 129.5, 129.8, 130.6, 132.4, 132.6, 137.6, 140.9, 156.8, 170.4, 175.1, 195.1153. *t*_R,LCMS_ = 2.45 min; MS (ESI^+^) *m/z* 492.3 [M + H]^+^. Anal. Calcd (%) for C_27_H_25_NO_6_S: C, 65.97; H, 5.13; N, 2.85; found C, 65.77; H, 5.31; N, 2.68.

**2.1.39. 3-{4-[2–(6-benzoyl-2-oxo-benzothiazol-3-yl)-ethoxy]-phenyl}-2–(2,2,2-trifluoro)ethoxy-propionic acid (32 b)**

Off-white solid; 74% yield; C_27_H_22_F_3_NO_6_S; MW = 545.52 g/mol; m.p. 60–62 °C. ^1^H NMR (CDCl_3_): *δ* 2.99 (dd, 1H, *J* = 7.8 Hz, 14.65 Hz); 3.11 (dd, 1H, *J* = 4.25 Hz, 14.65 Hz), 3.71 (m, 1H); 4.00 (m, 1H); 4.20 (dd, 1H, *J* = 4.25 Hz, 7.8 Hz); 4.31 (t, 2H, *J* = 5.1 Hz); 4.40 (t, 2H, *J* = 5.1 Hz); 6.74 (d, 2H, *J* = 8.65 Hz); 7.13 (d, 2H, *J* = 8.65 Hz); 7.38 (d, 1H, *J* = 8.55 Hz); 7.51 (t, 2H, *J* = 7.45 Hz); 7.62 (t, 1H, *J* = 7.45 Hz); 7.78 (d, 2H, *J* = 7.45 Hz); 7.85 (dd, 1H, *J* = 1.7 Hz, 8.55 Hz); 7.92 (d, 1H, *J* = 1.7 Hz); ^13 ^C NMR (75 MHz, CDCl_3_): *δ* 37.7, 42.9, 65.4, 68.05 (q, *J*_C-F_ = 34.8 Hz), 77.2, 80.7, 111.0, 114.2, 122.6, 124.9, 128.4, 128.6, 129.0, 129.9, 130.6, 132.5, 132.6, 137.5, 140.9, 157.0, 170.5, 173.9, 195.3. *t*_R,LCMS_ = 2.50 min; MS (ESI^+^) *m/z* 546.3 [M + H]^+^. Anal. Calcd (%) for C_27_H_22_F_3_NO_6_S: C, 59.45; H, 4.06; N, 2.57; found C, 59.56; H, 3.98; N, 2.49.

**2.1.40. 3-{4-[2–(6-benzoyl-3-oxo-benzo[1, 4]thiazin-4-yl)-ethoxy]-phenyl}-2-ethoxypropionic acid (33)**

Pale yellow solid; 78% yield; C_28_H_27_NO_6_S; MW= 505.59 g/mol; m.p. 52–54 °C. ^1^H NMR (CDCl_3_): *δ* 1.16 (t, 3H, *J* = 7 Hz); 2.92 (dd, 1H, *J* = 7.5 Hz, 14.2 Hz); 3.06 (dd, 1H, *J* = 4.25 Hz, 14.2 Hz); 3.45 (m, 3H); 3.58 (m, 1H); 4.03 (dd, 1H, *J* = 4.25 Hz, 7.5 Hz); 4.28 (t, 2H, *J* = 4.6 Hz); 4.35 (t, 2H, *J* = 4.6 Hz); 6.69 (d, 2H, *J* = 8.7 Hz); 7.11 (d, 2H, *J* = 8.7 Hz); 7.46–7.52 (m, 4H); 7.61 (t, 1H, *J* = 7.7 Hz); 7.82 (d, 2H, *J* = 7.3 Hz); 8.22 (d, 1H, *J* = 1.5 Hz); ^13 ^C NMR (75 MHz, CDCl_3_): *δ* 15.0, 31.4, 37.7, 46.8, 65.6, 66.8, 79.7, 114.3, 120.7, 125.4, 127.7, 128.4, 129.1, 129.8, 129.9, 130.5, 132.7, 136.6, 137.3, 140.5, 157.1, 164.9, 174.0, 195.4. *t*_R,LCMS_ = 2.42 min; MS (ESI^+^) *m/z* 506.3 [M + H]^+^. Anal. Calcd (%) for C_28_H_27_NO_6_S: C, 66.52; H, 5.38; N, 2.77; found C, 66.88; H, 5.74; N, 2.51.

**2.1.41. 3-{4-[2–(7-benzoyl-3-oxo-benzo[1, 4]thiazin-4-yl)-ethoxy]-phenyl}-2-ethoxypropionic acid (34)**

Off-white solid; 79% yield; C_28_H_27_NO_6_S; MW = 505.59 g/mol; m.p. 58–60 °C. ^1^H NMR (CDCl_3_): *δ* 1.18 (t, 3H, *J* = 6.95 Hz); 2.95 (dd, 1H, *J* = 7.45 Hz, 14.5 Hz); 3.08 (dd, 1H, *J* = 4.4 Hz, 14.5 Hz); 3.45 (m, 3H); 3.60 (m, 1H); 4.04 (dd, 1H, *J* = 4.4 Hz, 7.45 Hz); 4.32 (t, 2H, *J* = 5 Hz); 4.39 (t, 2H, *J* = 5 Hz); 6.81 (d, 2H, *J* = 8.6 Hz); 7.15 (d, 2H, *J* = 8.6 Hz); 7.51 (t, 2H, *J* = 7.75 Hz); 7.62 (t, 1H, *J* = 7.45 Hz); 7.68 (d, 1H, *J* = 8.7 Hz); 7.74 (dd, 1H, *J* = 2 Hz, 8.7 Hz); 7.79 (d, 2H, *J* = 8.25 Hz); 7.85 (d, 1H, *J* = 2 Hz); ^13 ^C NMR (75 MHz, CDCl_3_): *δ* 15.0, 31.3, 37.6, 46.3, 65.3; 66.8, 79.6, 114.3, 118.6, 124.0, 128.4, 129.2, 129.4, 129.8, 130.1, 130.6, 132.6, 132.7, 137.3, 143.4, 157.1, 165.1, 173.9, 194.8. *t*_R,LCMS_ = 2.44 min; MS (ESI^+^) *m/z* 506.3 [M + H]^+^. Anal. Calcd (%) for C_28_H_27_NO_6_S: C, 66.52; H, 5.38; N, 2.77; found C, 66.53; H, 5.31; N, 2.54.

**2.1.42. 3-{4-[2–(6-benzoyl-3-oxo-benzo[1, 4]oxazin-4-yl)-ethoxy]-phenyl}-2-ethoxypropionic acid (35)**

Off-white solid; 76% yield; C_28_H_27_NO_7_; MW = 489.53 g/mol; m.p. 47–49 °C. ^1^H NMR (CDCl_3_): *δ* 1.17 (t, 3H, *J* = 7.5 Hz); 2.94 (m, 1H); 3.04 (m, 1H); 3.45 (m, 1H); 3.58 (m, 1H); 4.02 (dd, 1H, *J* = 4.25 Hz, 7.4 Hz); 4.26 (t, 2H, *J* = 5 Hz); 4.36 (t, 2H, *J* = 5 Hz); 4.70 (s, 2H); 6.75 (d, 2H, *J* = 8.65 Hz); 7.04 (d, 1H, *J* = 8.2 Hz); 7.12 (d, 2H, *J* = 8.65 Hz); 7.45–7.52 (m, 3H); 7.59 (m, 1H); 7.79 (d, 2H, *J* = 8.55 Hz); 8.00 (d, 1H, *J* = 1.75 Hz); ^13 ^C NMR (75 MHz, CDCl_3_): *δ* 15.0, 29.7, 37.7, 42.0, 65.2, 67.6, 79.7, 114.2, 114.3, 116.4, 118.0, 127.4, 128.3, 129.8, 129.8, 130.5, 130.6, 132.4, 137.6, 148.9, 157.1, 164.0, 173.9, 195.1. *t*_R,LCMS_ = 2.30 min; MS (ESI^+^) *m/z* 490.3 [M + H]^+^. Anal. Calcd (%) for C_28_H_27_NO_7_: C, 68.70; H, 5.56; N, 2.86; found C, 68.53; H, 5.31; N, 2.63.

**2.1.43. 3-{4-[2–(7-benzoyl-3-oxo-benzo[1, 4]oxazin-4-yl)-ethoxy]-phenyl}-2-ethoxypropionic acid (36)**

Off-white solid; 76% yield; C_28_H_27_NO_7_; MW = 489.51 g/mol; m.p. 36–38 °C. ^1^H NMR (CDCl_3_): *δ* 1.17 (t, 3H, *J* = 7 Hz); 2.95 (dd, 1H, *J* = 7.75 Hz, 14.25 Hz); 3.06 (dd, 1H, *J* = 4.5 Hz, 14.25 Hz); 3.43 (m, 1H), 3.62 (m, 1H), 4.03 (dd, 1H, *J* = 4.5 Hz, 7.75 Hz); 4.29 (t, 2H, *J* = 4.9 Hz); 4.38 (t, 2H, *J* = 4.9 Hz); 4.68 (s, 2H); 6.79 (d, 2H, *J* = 8.65 Hz); 7.16 (d, 2H, *J* = 8.65 Hz); 7.44 (d, 1H, *J* = 8.4 Hz); 7.48–7.53 (m, 3H); 7.57 (dd, 1H, *J* = 1.9 Hz, 8.4 Hz); 7.61 (t, 1H, *J* = 7.6 Hz); 7.90 (dd, 2H, *J* = 1.6 Hz, 8.4 Hz); ^13 ^C NMR (75 MHz, CDCl_3_): *δ* 15.0, 37.8, 42.0, 65.0, 66.7, 67.5, 79.6, 114.2, 114.4, 115.6,118.8, 125.4, 128.3, 129.8, 130.6, 132.4, 132.9, 133.3, 137.4, 144.8, 157.0, 164.7, 175.4, 195.0. *t*_R,LCMS_ = 2.34 min; MS (ESI^+^) *m/z* 490.3 [M + H]^+^. Anal. Calcd (%) for C_28_H_27_NO_7_: C, 68.70; H, 5.56; N, 2.86; found C, 68.54; H, 5.63; N, 2.57.

**2.1.44. 3-{4-[2–(5-benzoyl-2-oxo-2,3-dihydroindol-1-yl)ethoxy]phenyl}-2-ethoxypropionic acid (37)**

Pale yellow solid; 73% yield; C_28_H_27_NO_6_; MW = 473.51 g/mol; m.p. 68–70 °C. 1H NMR (CDCl_3_): *δ* 1.18 (t, 3H, *J* = 6.95 Hz); 2.63 (s, 2H); 2.95 (dd, 1H, *J* = 7.05 Hz, 14.1 Hz); 3.07 (dd, 1H, *J* = 4.3 Hz, 14.1 Hz); 3.48 (m, 1H); 3.59 (m, 1H); 4.05 (dd, 1H, *J* = 4.03 Hz, 7.05 Hz); 4.20 (t, 2H, *J* = 5.05 Hz); 4.28 (t, 2H, *J* = 5.05 Hz); 6.75 (d, 2H, *J* = 8.65 Hz); 7.14 (d, 2H, *J* = 8.65 Hz); 7.33 (d, 1H, *J* = 8.4 Hz); 7.52 (t, 2H, *J* = 7.65 Hz); 7.64 (t, 1H, *J* = 7.2 Hz); 7.75 (d, 2H, *J* = 7.95 Hz); 8.01 (d, 1H, *J* = 1.8 Hz); 8.24 (dd, 1H, *J* = 1.8 Hz, 8.4 Hz); ^13 ^C NMR (75 MHz, CDCl_3_): *δ* 15.1, 37.4, 40.5, 40.8, 65.72, 66.9, 79.5, 111.4, 114.2, 116.8, 127.2, 128.6, 129.4, 129.6, 130.7, 132.8, 136.9, 140.3, 154.4, 156.8, 158.5, 172.8, 182.2, 194.2. *t*_R,LCMS_ = 2.25 min; MS (ESI^+^) *m/z* 474.3 [M + H]^+^. Anal. Calcd (%) for C_28_H_27_NO_6_: C, 71.02; H, 5.75; N, 2.96; found C, 71.19; H, 5.59; N, 2.90.

**2.1.45. 3-{4-[2–(5-benzoyl-3,3-difluoro-2-oxo-2,3-dihydroindol-1-yl)ethoxy]phenyl}-2-ethoxy-propionic acid (38)**

Pale yellow solid; 84% yield; C_28_H_25_F_2_NO_6_; MW = 509.49 g/mol; m.p. 78–79 °C. 1H NMR (CDCl_3_): *δ* 1.14 (t, 3H, *J* = 6.95 Hz); 2.92 (dd, 1H, *J* = 7.35 Hz, 14.05 Hz); 3.05 (dd, 1H, *J* = 4.35 Hz, 14.05 Hz); 3.43 (m, 1H); 3.59 (m, 1H); 4.01 (dd, 1H, *J* = 4.35 Hz, 7.35 Hz); 4.15 (t, 2H, *J* = 4.9 Hz); 4.24 (t, 2H, *J* = 4.9 Hz); 6.73 (d, 2H, *J* = 8.65 Hz); 7.13 (d, 2H, *J* = 8.65 Hz); 7.29 (m, 1H); 7.52 (t, 2H, *J* = 7.95 Hz); 7.63 (t, 1H, *J* = 7.3 Hz); 7.77 (d, 2H, *J* = 6.9 Hz); 8.01 (d, 1H, *J* = 1.8 Hz); 8.06 (dd, 1H, *J* = 1.8 Hz, 8.55 Hz); ^13 ^C NMR (75 MHz, CDCl_3_): *δ* 15.0, 37.6, 40.6, 65.4, 66.7, 79.8, 110.0, 110.6, 114.2, 119.9, 126.6, 128.5, 129.7, 130.6, 132.7, 133.4, 136.2, 137.1, 147.4, 156.7, 165.7, 174.6, 194.4. *t*_R,LCMS_ = 2.49 min; MS (ESI^+^) *m/z* 510.3 [M + H]^+^. Anal. Calcd (%) for C_28_H_25_F_2_NO_6_: C, 66.01; H, 4.95; N, 2.75; found C, 66.22; H, 5.01; N, 2.69.

**2.1.46. General procedure for synthesis of optically pure carboxylic acids (41a,b and 42a,b)**

A solution of **2** (0.5 g, 2 mmol, 1.0 eq) in HMPA (5 mL) was added dropwise to a 60% NaH dispersion in mineral oil (0.12 g, 3 mmol, 1.5 eq) in HMPA (5 mL) at 0 °C under N_2_, and the solution was stirred for 30 min at room temperature. A solution of **21a** or **22a** (0.66 g, 2.4 mmol, 1.2 eq) or **21 b** or **22 b** (0.78 g, 2.4 mmol, 1.2 eq) in HMPA (5 mL) was then added, and the mixture was stirred at room temperature for 12 h under N_2_. The reaction mixture was hydrolysed with 1 M aqueous HCl solution, and the precipitate was filtered off, dried and purified by flash column chromatography (DCM/MeOH 95:5).

**2.1.47. (S)-3-{4-[2–(6-benzoyl-2-oxo-benzothiazol-3-yl)-ethoxy]-phenyl}-2-ethoxypropionic acid (41a)**

Off-White solid; 79% yield; C_27_H_25_NO_6_S; MW= 491.57 g/mol; m.p. 57–59 °C. ^1^H NMR (CDCl_3_): *δ* 1.16 (t, 3H, *J* = 6.95 Hz); 2.93 (dd, 1H, *J* = 7.55 Hz, 14.05 Hz); 3.04 (dd, 1H, *J* = 4.4 Hz, 14.05 Hz); 3.41 (m, 1H); 3.60 (m, 1H); 4.02 (dd, 1H, *J* = 4.4 Hz, 7.65 Hz); 4.30 (t, 2H, *J* = 5.45 Hz); 4.40 (t, 2H, *J* = 5.45 Hz); 6.74 (d, 2H, *J* = 8.5 Hz); 7.13 (d, 2H, *J* = 8.5 Hz); 7.41 (d, 1H, *J* = 8.6 Hz); 7.51 (t, 2H, *J* = 7.8 Hz); 7.62 (t, 1H, *J* = 7.5 Hz); 7.78 (d, 2H, *J* = 8 Hz); 7.86 (dd, 1H, *J* = 1.8 Hz, 8.5 Hz); 7.92 (d, 1H, *J* = 1.8 Hz). ^13 ^C NMR (75 MHz, CDCl_3_): *δ* 15.0, 37.7, 42.9, 65.5, 66.7, 79.6, 111.1, 114.2, 122.6, 124.8, 128.4, 129.0, 129.5, 129.8, 130.6, 132.4, 132.6, 137.6, 140.9, 156.8, 170.4, 175.1, 195.1153. *t*_R,LCMS_ = 2.45 min; MS (ESI^+^) *m/z* 492.3 [M + H]^+^. Chiral HPLC method: flow rate= 1.5 mL/min, eluents = A/B 2:8, tr = 25.44 min.

**2.1.48. (S)-3-{4-[2–(6-benzoyl-2-oxo-benzothiazol-3-yl)-ethoxy]-phenyl}-2–(2,2,2-trifluoro)ethoxy-propionic acid (41 b)**

Off-white solid; 76% yield; C_27_H_22_F_3_NO_6_S; MW= 545.52 g/mol; m.p. 60–62 °C. ^1^H NMR (CDCl_3_): *δ* 2.99 (dd, 1H, *J* = 7.8 Hz, 14.65 Hz); 3.11 (dd, 1H, *J* = 4.25 Hz, 14.65 Hz), 3.71 (m, 1H); 4.00 (m, 1H); 4.20 (dd, 1H, *J* = 4.25 Hz, 7.8 Hz); 4.31 (t, 2H, *J* = 5.1 Hz); 4.40 (t, 2H, *J* = 5.1 Hz); 6.74 (d, 2H, *J* = 8.65 Hz); 7.13 (d, 2H, *J* = 8.65 Hz); 7.38 (d, 1H, *J* = 8.55 Hz); 7.51 (t, 2H, *J* = 7.45 Hz); 7.62 (t, 1H, *J* = 7.45 Hz); 7.78 (d, 2H, *J* = 7.45 Hz); 7.85 (dd, 1H, *J* = 1.7 Hz, 8.55 Hz); 7.92 (d, 1H, *J* = 1.7 Hz); ^13 ^C NMR (75 MHz, CDCl_3_): *δ* 37.7, 42.9, 65.4, 68.05 (q, *J*_C–F_ = 34.8 Hz), 77.2, 80.7, 111.0, 114.2, 122.6, 124.9, 128.4, 128.6, 129.0, 129.9, 130.6, 132.5, 132.6, 137.5, 140.9, 157.0, 170.5, 173.9, 195.3. *t*_R,LCMS_ = 2.50 min; MS (ESI^+^) *m/z* 546.3 [M + H]^+^. Chiral HPLC method: flow rate= 1.5 mL/min, eluents = A/B 2:8, tr = 22.36 min.

**2.1.49. (R)-3-{4-[2–(6-benzoyl-2-oxo-benzothiazol-3-yl)-ethoxy]-phenyl}-2-ethoxypropionic acid (42a)**

Off-White solid; 72% yield; C_27_H_25_NO_6_S; MW= 491.57 g/mol; m.p. 57–59 °C. ^1^H NMR (CDCl_3_): *δ* 1.16 (t, 3H, *J* = 6.95 Hz); 2.93 (dd, 1H, *J* = 7.55 Hz, 14.05 Hz); 3.04 (dd, 1H, *J* = 4.4 Hz, 14.05 Hz); 3.41 (m, 1H); 3.60 (m, 1H); 4.02 (dd, 1H, *J* = 4.4 Hz, 7.65 Hz); 4.30 (t, 2H, *J* = 5.45 Hz); 4.40 (t, 2H, *J* = 5.45 Hz); 6.74 (d, 2H, *J* = 8.5 Hz); 7.13 (d, 2H, *J* = 8.5 Hz); 7.41 (d, 1H, *J* = 8.6 Hz); 7.51 (t, 2H, *J* = 7.8 Hz); 7.62 (t, 1H, *J* = 7.5 Hz); 7.78 (d, 2H, *J* = 8 Hz); 7.86 (dd, 1H, *J* = 1.8 Hz, 8.5 Hz); 7.92 (d, 1H, *J* = 1.8 Hz). ^13 ^C NMR (75 MHz, CDCl_3_): *δ* 15.0, 37.7, 42.9, 65.5, 66.7, 79.6, 111.1, 114.2, 122.6, 124.8, 128.4, 129.0, 129.5, 129.8, 130.6, 132.4, 132.6, 137.6, 140.9, 156.8, 170.4, 175.1, 195.1153. *t*_R,LCMS_ = 2.45 min; MS (ESI^+^) *m/z* 492.3 [M + H]^+^. Chiral HPLC method: flow rate = 1.5 mL/min, eluents = A/B 2:8, tr = 22.38 min.

**2.1.50. (R)-3-{4-[2–(6-benzoyl-2-oxo-benzothiazol-3-yl)-ethoxy]-phenyl}-2–(2,2,2-trifluoro)ethoxy-propionic acid (42 b)**

Off-white solid; 74% yield; C_27_H_22_F_3_NO_6_S; MW= 545.52 g/mol; m.p. 60–62 °C. ^1^H NMR (CDCl_3_): *δ* 2.99 (dd, 1H, *J* = 7.8 Hz, 14.65 Hz); 3.11 (dd, 1H, *J* = 4.25 Hz, 14.65 Hz), 3.71 (m, 1H); 4.00 (m, 1H); 4.20 (dd, 1H, *J* = 4.25 Hz, 7.8 Hz); 4.31 (t, 2H, *J* = 5.1 Hz); 4.40 (t, 2H, *J* = 5.1 Hz); 6.74 (d, 2H, *J* = 8.65 Hz); 7.13 (d, 2H, *J* = 8.65 Hz); 7.38 (d, 1H, *J* = 8.55 Hz); 7.51 (t, 2H, *J* = 7.45 Hz); 7.62 (t, 1H, *J* = 7.45 Hz); 7.78 (d, 2H, *J* = 7.45 Hz); 7.85 (dd, 1H, *J* = 1.7 Hz, 8.55 Hz); 7.92 (d, 1H, *J* = 1.7 Hz); ^13 ^C NMR (75 MHz, CDCl_3_): *δ* 37.7, 42.9, 65.4, 68.05 (q, *J*_C–F_ = 34.8 Hz), 77.2, 80.7, 111.0, 114.2, 122.6, 124.9, 128.4, 128.6, 129.0, 129.9, 130.6, 132.5, 132.6, 137.5, 140.9, 157.0, 170.5, 173.9, 195.3. *t*_R,LCMS_ = 2.50 min; MS (ESI^+^) *m/z* 546.3 [M + H]^+^. Chiral HPLC method: flow rate = 1.5 mL/min, eluents = A/B 2:8, tr = 17.10 min.

**2.1.51. General procedure for the synthesis of oxime 39a,b, 43a, 45a and O-methyl oxime ethers 40a,b, 44a,b, 46a,b**

Hydroxylamine hydrochloride (0.69 g, 10 mmol, 4.0 eq) or *O*-methylhydroxylamine hydrochloride (0.83 g, 10 mmol, 4 equiv) was added to a solution of **32a,b**, **41a,b** or **42a,b** (2.5 mmol, 1.0 eq) in pyridine (20 mL), and the mixture was stirred at 100 °C for 4 h. The reaction mixture was concentrated *in vacuo*, hydrolysed with 1 M aqueous HCl solution, and extracted twice with DCM. The combined organic extracts were dried over MgSO4, filtered off, evaporated under reduced pressure, and the resulting residues were purified by flash column chromatography (toluene/EtOAc 97:3).

**2.1.52. 3-{4-[2–(6-[(E)-hydroxyimino-phenyl-methyl]-2-oxo-benzothiazol-3-yl)-ethoxy]-phenyl}-2-ethoxypropionic acid (39a)**

Off-white solid; 76% yield; C_27_H_26_N_2_O_6_S; MW= 506.57 g/mol; m.p. 84–85 °C. ^1^H NMR (CDCl_3_): *δ* 1.17 (t, 3H, *J* = 6.9 Hz); 2.95 (dd, 1H, *J* = 7.5 Hz, 14.2 Hz); 3.06 (dd, 1H, *J* = 4.5 Hz, 14.2 Hz); 3.43 (m, 1H); 3.61 (m, 1H); 4.04 (dd, 1H, *J* = 4.5 Hz, 7.5 Hz); 4.28 (t, 2H, *J* = 4.7 Hz); 4.35 (t, 2H, *J* = 4.7 Hz); 6.76 (d, 2H, *J* = 8.6 Hz); 7.14 (d, 2H, *J* = 8.6 Hz); 7.30 (d, 1H, *J* = 8.45 Hz); 7.39–7.52 (m, 7H); ^13 ^C NMR (75 MHz, CDCl_3_): *δ* 15.0, 37.8, 42.7, 65.5, 66.7, 79.7, 111.4, 114.2, 122.2, 122.7, 126.0, 128.4, 129.3, 129.4, 129.5, 130.6, 131.4, 132.1, 138.6, 156.9, 157.1, 170.3, 174.8. *t*_R,LCMS_ = 2.25 min; MS (ESI^+^) *m/z* 507.3 [M + H]^+^. Anal. Calcd (%) for C_27_H_26_N_2_O_6_S: C, 64.02; H, 5.17; N, 5.53; found C, 64.21; H, 5.21; N, 5.44.

**2.1.53. 3-{4-[2–(6-[(Z/E)-hydroxyimino-phenyl-methyl]-2-oxo-benzothiazol-3-yl)-ethoxy]-phenyl}-2–(2,2,2-trifluoro)ethoxypropionic acid (39 b)**

Off-white solid; 74% yield; C_27_H_23_F_3_N_2_O_6_S; MW = 560.54 g/mol; m.p. 55–56 °C. ^1^H NMR (CDCl_3_): *δ* 3.06 (m, 2H); 3.69 (m, 1H); 4.01 (m, 1H); 4.21 (m, 1H); 4.28–4.39 (m, 4H); 5.67 (br s, 1H); 6.75 (m, 2H); 7.15 (m, 2H); 7.35–7.62 (m, 7H); 7.77–7.92 (m, 1H); ^13 ^C NMR (75 MHz, CDCl_3_): *δ* 37.9, 42.7, 65.4, 67.2, 67.7, 68.1, 68.4, 68.6, 80.9, 111.0, 111.4, 114.2, 114.3, 122.3, 122.5, 123.9, 124.9, 126.0, 128.2, 128.4, 128.5, 129.4, 129.8, 129.9, 130.6, 135.7, 137.5, 138.2, 138.7, 140.9, 157.0, 170.3, 174.5. *t*_R,LCMS_ = 2.32 min; MS (ESI^+^) *m/z* 561.3 [M + H]^+^. Anal. Calcd (%) for C_27_H_23_F_3_N_2_O_6_S: C, 57.85; H, 5.14; N, 5.00; found C, 57.95; H, 5.03; N, 5.12.

**2.1.54. 3-{4-[2–(6-[(E)-methoxyimino-phenyl-methyl]-2-oxo-benzothiazol-3-yl)-ethoxy]-phenyl}-2-ethoxypropionic acid (40a)**

Off-white solid; 73% yield; C_28_H_28_N_2_O_6_S; MW= 520.59 g/mol; m.p. 151–152 °C. ^1^H NMR (CDCl_3_): *δ* 1.18 (t, 3H, *J* = 7 Hz); 2.94 (dd, 1H, *J* = 7.6 Hz, 14.4 Hz); 3.07 (dd, 1H, *J* = 4.3 Hz, 14.4 Hz); 3.46 (m, 1H); 3.60 (m, 1H); 4.00 (s, 3H); 4.04 (dd, 1H, *J* = 4.3 Hz, 7.6 Hz); 4.28 (t, 2H, *J* = 5 Hz); 4.35 (t, 2H, *J* = 5 Hz); 6.75 (d, 2H, *J* = 8.8 Hz); 7.13 (d, 2H, *J* = 8.8 Hz); 7.25 (d, 1H, *J* = 8.6 Hz); 7.36 (m, 2H); 7.44–7.51 (m, 4H); 7.57 (d, 1H, *J* = 1.7 Hz); ^13^C NMR (75 MHz, CDCl_3_): *δ* 15.0, 37.6, 42.7, 62.5, 65.4, 66.9, 79.6, 111.1, 114.2, 121.8, 122.6, 126.1, 128.3, 129.1, 129.1, 129.2, 130.6, 131.8, 132.9, 138.3, 155.8, 157.0, 170.4, 174.1. *t*_R,LCMS_ = 2.7 min; MS (ESI^+^) *m/z* 521.3 [M + H]^+^. Anal. Calcd (%) for C_28_H_28_N_2_O_6_S: C, 64.60; H, 5.42; N, 5.38; found C, 64.71; H, 5.31; N, 5.46.

**2.1.55. 3-{4-[2–(6-[(E)-methoxyimino-phenyl-methyl]-2-oxo-benzothiazol-3-yl)-ethoxy]-phenyl}-2–(2,2,2-trifluoro)ethoxypropionic acid (40 b)**

Off-white solid; 81% yield; C_28_H_25_F_3_N_2_O_6_S; MW = 574.56 g/mol; m.p. 56–57 °C. ^1^H NMR (CDCl_3_): *δ* 3.01 (dd, 1H, *J* = 7.65 Hz, 14.4 Hz); 3.13 (dd, 1H, *J* = 4.15 Hz, 14.4 Hz); 3.73 (m, 1H), 4.00 (m, 4H), 4.22 (dd, 1H, *J* = 4.15 Hz, 7.65 Hz); 4.30 (t, 2H, *J* = 4.3 Hz); 4.35 (t, 2H, *J* = 4.3 Hz); 6.75 (d, 2H, *J* = 8.7 Hz); 7.13 (d, 2H, *J* = 8.7 Hz); 7.24 (d, 1H, *J* = 8.7 Hz); 7.37 (m, 2H); 7.44–7.51 (m, 4H); 7.57 (d, 1H, *J* = 1.7 Hz); ^13 ^C NMR (75 MHz, CDCl_3_): *δ* 37.8, 42.7, 62.5, 65.3, 68.0 (q, *J*_C–F_ = 34.45 Hz), 77.2, 80.8, 111.0, 114.3, 121.9, 122.6, 126.1, 128.0, 128.3, 128.3, 128.5, 129.1, 130.6, 131.7, 132.9, 138.3, 155.9, 157.1, 170.4, 173.9. *t*_R,LCMS_ = 2.67 min; MS (ESI^+^) *m/z* 575.3 [M + H]^+^. Anal. Calcd (%) for C_28_H_25_F_3_N_2_O_6_S: C, 58.53; H, 4.39; N, 4.88; found C, 58.69; H, 4.25; N, 4.78.

**2.1.56. (S)-3-{4-[2–(6-[(E)-hydroxyimino-phenyl-methyl]-2-oxo-benzothiazol-3-yl)-ethoxy]-phenyl}-2-ethoxypropionic acid (43a)**

Off-white solid; 78% yield; C_27_H_26_N_2_O_6_S; MW= 506.57 g/mol; m.p. 84–85 °C. ^1^H NMR (CDCl_3_): *δ* 1.17 (t, 3H, *J* = 6.9 Hz); 2.95 (dd, 1H, *J* = 7.5 Hz, 14.2 Hz); 3.06 (dd, 1H, *J* = 4.5 Hz, 14.2 Hz); 3.43 (m, 1H); 3.61 (m, 1H); 4.04 (dd, 1H, *J* = 4.5 Hz, 7.5 Hz); 4.28 (t, 2H, *J* = 4.7 Hz); 4.35 (t, 2H, *J* = 4.7 Hz); 6.76 (d, 2H, *J* = 8.6 Hz); 7.14 (d, 2H, *J* = 8.6 Hz); 7.30 (d, 1H, *J* = 8.45 Hz); 7.39–7.52 (m, 7H); ^13 ^C NMR (75 MHz, CDCl_3_): *δ* 15.0, 37.8, 42.7, 65.5, 66.7, 79.7, 111.4, 114.2, 122.2, 122.7, 126.0, 128.4, 129.3, 129.4, 129.5, 130.6, 131.4, 132.1, 138.6, 156.9, 157.1, 170.3, 174.8. *t*_R,LCMS_ = 2.25 min; MS (ESI^+^) *m/z* 507.3 [M + H]^+^. Chiral HPLC method: flow rate = 1.5 mL/min, eluents = A/B 2:8, tr = 32.19 min.

**2.1.57. (S)-3-{4-[2–(6-[(E)-methoxyimino-phenyl-methyl]-2-oxo-benzothiazol-3-yl)-ethoxy]-phenyl}-2-ethoxypropionic acid (44a)**

Off-white solid; 79% yield; C_28_H_28_N_2_O_6_S; MW = 520.59 g/mol; m.p. 151–152 °C. ^1^H NMR (CDCl_3_): *δ* 1.18 (t, 3H, *J* = 7 Hz); 2.94 (dd, 1H, *J* = 7.6 Hz, 14.4 Hz); 3.07 (dd, 1H, *J* = 4.3 Hz, 14.4 Hz); 3.46 (m, 1H); 3.60 (m, 1H); 4.00 (s, 3H); 4.04 (dd, 1H, *J* = 4.3 Hz, 7.6 Hz); 4.28 (t, 2H, *J* = 5 Hz); 4.35 (t, 2H, *J* = 5 Hz); 6.75 (d, 2H, *J* = 8.8 Hz); 7.13 (d, 2H, *J* = 8.8 Hz); 7.25 (d, 1H, *J* = 8.6 Hz); 7.36 (m, 2H); 7.44–7.51 (m, 4H); 7.57 (d, 1H, *J* = 1.7 Hz); ^13 ^C NMR (75 MHz, CDCl_3_): *δ* 15.0, 37.6, 42.7, 62.5, 65.4, 66.9, 79.6, 111.1, 114.2, 121.8, 122.6, 126.1, 128.3, 129.1, 129.1, 129.2, 130.6, 131.8, 132.9, 138.3, 155.8, 157.0, 170.4, 174.1. *t*_R,LCMS_ = 2.7 min; MS (ESI^+^) *m/z* 521.3 [M + H]^+^. Chiral HPLC method: flow rate= 1 mL/min, eluents = A/B 2:8, tr = 16.05 min.

**2.1.58. (S)-3-{4-[2–(6-[(E)-methoxyimino-phenyl-methyl]-2-oxo-benzothiazol-3-yl)-ethoxy]-phenyl}-2–(2,2,2-trifluoro)ethoxypropionic acid (44 b)**

Off-white solid; 81% yield; C_28_H_25_F_3_N_2_O_6_S; MW= 574.56 g/mol; m.p. 56–57 °C. ^1^H NMR (CDCl_3_): *δ* 3.01 (dd, 1H, *J* = 7.65 Hz, 14.4 Hz); 3.13 (dd, 1H, *J* = 4.15 Hz, 14.4 Hz); 3.73 (m, 1H), 4.00 (m, 4H), 4.22 (dd, 1H, *J* = 4.15 Hz, 7.65 Hz); 4.30 (t, 2H, *J* = 4.3 Hz); 4.35 (t, 2H, *J* = 4.3 Hz); 6.75 (d, 2H, *J* = 8.7 Hz); 7.13 (d, 2H, *J* = 8.7 Hz); 7.24 (d, 1H, *J* = 8.7 Hz); 7.37 (m, 2H); 7.44–7.51 (m, 4H); 7.57 (d, 1H, *J* = 1.7 Hz); ^13 ^C NMR (75 MHz, CDCl_3_): *δ* 37.8, 42.7, 62.5, 65.3, 68.0 (q, *J*_C–F_ = 34.45 Hz), 77.2, 80.8, 111.0, 114.3, 121.9, 122.6, 126.1, 128.0, 128.3, 128.3, 128.5, 129.1, 130.6, 131.7, 132.9, 138.3, 155.9, 157.1, 170.4, 173.9. *t*_R,LCMS_ = 2.67 min; MS (ESI^+^) *m/z* 575.3 [M + H]^+^. Chiral HPLC method: flow rate= 1 mL/min, eluents = A/B 15:85, tr = 22.02 min.

**2.1.59. (R)-3-{4-[2–(6-[(E)-hydroxyimino-phenyl-methyl]-2-oxo-benzothiazol-3-yl)-ethoxy]-phenyl}-2-ethoxypropionic acid (45a)**

Off-white solid; 72% yield; C_27_H_26_N_2_O_6_S; MW= 506.57 g/mol; m.p. 84–85 °C. ^1^H NMR (CDCl_3_): *δ* 1.17 (t, 3H, *J* = 6.9 Hz); 2.95 (dd, 1H, *J* = 7.5 Hz, 14.2 Hz); 3.06 (dd, 1H, *J* = 4.5 Hz, 14.2 Hz); 3.43 (m, 1H); 3.61 (m, 1H); 4.04 (dd, 1H, *J* = 4.5 Hz, 7.5 Hz); 4.28 (t, 2H, *J* = 4.7 Hz); 4.35 (t, 2H, *J* = 4.7 Hz); 6.76 (d, 2H, *J* = 8.6 Hz); 7.14 (d, 2H, *J* = 8.6 Hz); 7.30 (d, 1H, *J* = 8.45 Hz); 7.39–7.52 (m, 7H); ^13 ^C NMR (75 MHz, CDCl_3_): *δ* 15.0, 37.8, 42.7, 65.5, 66.7, 79.7, 111.4, 114.2, 122.2, 122.7, 126.0, 128.4, 129.3, 129.4, 129.5, 130.6, 131.4, 132.1, 138.6, 156.9, 157.1, 170.3, 174.8. *t*_R,LCMS_ = 2.25 min; MS (ESI^+^) *m/z* 507.3 [M + H]^+^. Chiral HPLC method: flow rate = 1.5 mL/min, eluents = A/B 2:8, tr = 29.42 min.

**2.1.60. (R)-3-{4-[2–(6-[(E)-methoxyimino-phenyl-methyl]-2-oxo-benzothiazol-3-yl)-ethoxy]-phenyl}-2-ethoxypropionic acid (46a)**

Off-white solid; 75% yield; C_28_H_28_N_2_O_6_S; MW= 520.59 g/mol; m.p. 151–152 °C. ^1^H NMR (CDCl_3_): *δ* 1.18 (t, 3H, *J* = 7 Hz); 2.94 (dd, 1H, *J* = 7.6 Hz, 14.4 Hz); 3.07 (dd, 1H, *J* = 4.3 Hz, 14.4 Hz); 3.46 (m, 1H); 3.60 (m, 1H); 4.00 (s, 3H); 4.04 (dd, 1H, *J* = 4.3 Hz, 7.6 Hz); 4.28 (t, 2H, *J* = 5 Hz); 4.35 (t, 2H, *J* = 5 Hz); 6.75 (d, 2H, *J* = 8.8 Hz); 7.13 (d, 2H, *J* = 8.8 Hz); 7.25 (d, 1H, *J* = 8.6 Hz); 7.36 (m, 2H); 7.44–7.51 (m, 4H); 7.57 (d, 1H, *J* = 1.7 Hz); ^13 ^C NMR (75 MHz, CDCl_3_): *δ* 15.0, 37.6, 42.7, 62.5, 65.4, 66.9, 79.6, 111.1, 114.2, 121.8, 122.6, 126.1, 128.3, 129.1, 129.1, 129.2, 130.6, 131.8, 132.9, 138.3, 155.8, 157.0, 170.4, 174.1. *t*_R,LCMS_ = 2.7 min; MS (ESI^+^) *m/z* 521.3 [M + H]^+^. Chiral HPLC method: flow rate= 1 mL/min, eluents = A/B 2:8, tr = 13.51 min.

**2.1.61. (R)-3-{4-[2–(6-[(E)-methoxyimino-phenyl-methyl]-2-oxo-benzothiazol-3-yl)-ethoxy]-phenyl}-2–(2,2,2-trifluoro)ethoxypropionic acid (46 b)**

Off-white solid; 74% yield; C_28_H_25_F_3_N_2_O_6_S; MW= 574.56 g/mol; m.p. 56–57 °C. ^1^H NMR (CDCl_3_): *δ* 3.01 (dd, 1H, *J* = 7.65 Hz, 14.4 Hz); 3.13 (dd, 1H, *J* = 4.15 Hz, 14.4 Hz); 3.73 (m, 1H), 4.00 (m, 4H), 4.22 (dd, 1H, *J* = 4.15 Hz, 7.65 Hz); 4.30 (t, 2H, *J* = 4.3 Hz); 4.35 (t, 2H, *J* = 4.3 Hz); 6.75 (d, 2H, *J* = 8.7 Hz); 7.13 (d, 2H, *J* = 8.7 Hz); 7.24 (d, 1H, *J* = 8.7 Hz); 7.37 (m, 2H); 7.44–7.51 (m, 4H); 7.57 (d, 1H, *J* = 1.7 Hz); ^13 ^C NMR (75 MHz, CDCl_3_): *δ* 37.8, 42.7, 62.5, 65.3, 68.0 (q, *J*_C-F_ = 34.45 Hz), 77.2, 80.8, 111.0, 114.3, 121.9, 122.6, 126.1, 128.0, 128.3, 128.3, 128.5, 129.1, 130.6, 131.7, 132.9, 138.3, 155.9, 157.1, 170.4, 173.9. *t*_R,LCMS_ = 2.67 min; MS (ESI^+^) *m/z* 575.3 [M + H]^+^. Chiral HPLC method: flow rate= 1 mL/min, eluents = A/B 15:85, tr = 15.61 min.

### 2.2. Biological assay methods

**2.2.1. Membrane-bound PPARγ binding assay**

Binding assays were performed in 96-well plates format, using a classical filtration assay with a human full length PPARγ construct (GST-PPAR LBD (25 µg/mL)) expressed in bacteria with some modifications regarding the conditions of the experiments. The membrane-associated PPARγ was used as the biological source as previously described. Binding buffer consisted of 10 mM Tris/HCl, pH 8.2, containing 50 mM KCl and 1 mM dithiothreitol. Membrane preparations (5 µg/mL) were incubated for 180 min at 4 °C in the presence of [^3^H]rosiglitazone [BRL49653, Amersham] (4 nM) and the tested compounds. Non-specific binding was defined using an excess of unlabelled rosiglitazone (10 µM). Incubation was terminated by the addition of ice cold 50 mM Tris/HCl buffer pH 7.4 followed by rapid filtration under reduced pressure through Whatman GF/C filter plates pre-soaked with ice-cold buffer, followed by three successive washes with the same buffer. Radioactivity was measured in a TopCount apparatus (Packard). The receptor preparation used during these experiments presented a Bmax of 49 pmol/mg proteins and a Kd of 5.58 nM for [^3^H]-rosiglitazone. The compounds were solubilised in pure DMSO and diluted to the appropriate working concentrations (100 µM to 0.1 nM). For each compound tested, plots of ligand concentration versus DPM of radioligand bound were constructed and apparent Ki values were estimated from nonlinear least-squares fit of the data assuming simple competitive binding. The details of this assay have been reported^18^.

**2.2.2. Gene reporter assays: cell culture and transfection**

Cos-7 cells were transiently transfected with luciferase reporter plasmid (pG5-TK-pGL3) in the presence of pGal4-hPPARα or pGal4-hPPARγ (these vectors express chimeric proteins containing the Gal4 DNA-binding domain fused to the human PPARα or PPARγ ligand binding domain coding sequence) expression vector. Plasmids pGal4-hPPARα and γ pG5-TK-pGL3 were constructed as described previously^19^. Cells were seeded in 60 mm dishes at a density of 5.5 × 10^5^ cells/dish in DMEM supplemented with 10% FCS and incubated at 37 °C for 24 h prior to transfection. Cells were transfected in OptiMEM without FCS for 3 h at 37°, using polyethylenimine (PEI), with reporter and expression plasmids, as stated in figure’s legend. The plasmid pBluescript (Stratagene, La Jolla, CA) was used as carrier DNA to set the final amount of DNA to 5.5 µg/dish. The pCMV- ß-galactosidase expression plasmid was cotransfected as a control for transfection efficiency. Transfection was stopped by addition of DMEM supplemented with 10% FCS and cells were then incubated at 37 °C. After 16 h, cells were trypsinised and seeded in 96-well plates at the density of 2 × 10^4^ cells/well and incubated for 6 h in 10% FCS containing DMEM. Cells were then incubated for 24 h in DMEM containing 0.2% FCS and increasing concentrations of the compound tested or vehicle (DMSO). At the end of the experiment, cells were washed once with ice-cold PBS and the luciferase and the β–galactosidase assays were performed as described previously^20^. Cells were incubated 24 h in the presence of indicated concentrations of compound. Luciferase activity was measured and normalised to internal control β-galactosidase activity. Compounds which elicited on average at least 80% activation of PPARα or PPARγ versus respectively WY 14,643 and rosiglitazone (positive controls) were considered full agonists. EC_50_ were estimated using Prism software (GraphPad). All transfection experiments were performed at least three times.

**2.2.3. In vivo *ob/ob mice***

The glucose- and triglyceride-lowering activities of the compounds prepared were then tested using 10–12-week-old male ob/ob mice. The mice were housed in a temperature-controlled room (21.0–24.0 °C) at a relative humidity of 45–65%, and a 12 h light–dark cycle (light period 5:30–17:30). Mice had access to filtered (0.22 mm) tap water *ad libitum* and irradiated pelleted laboratory chow (transbreed, Special Diet Services, England) throughout the study. Compounds were prepared as a suspension in 1% hydroxyethylcellulose (HEC) for oral administration (10 mLkg^−^1). The mice (*n* = 8–12 per group) were dosed by gavage daily between 15:00 and 17:00 for 4 days. Randomisation was performed based on glycaemia values. The body weight gain (DBW in grams) of the animals was determined by measuring the difference between body weight at the beginning and end of the study for each mouse. At day 5 between 9:00 and 11:00 the mice were weighed, and blood samples were collected into heparin-containing tubes by retro-orbital puncture (500 mL per mouse). All procedures complied with International European Ethical Standards (86/609-EEC), the French National Committee (décret 87/848) for the care and use of laboratory animals and were approved by our institutional ethical committee. Plasma samples were prepared by centrifugation (2000 g for 10 min), and stored at −20 °C. For triglycerides and insulin determination, the percent change in plasma level in the treated group was calculated relative to the mean plasma level in the vehicle-treated group. ANOVA, followed by Dunnett’s comparison test (one-tailed) was used to estimate significant differences between the plasma glycaemia, triglycerides, insulin, and DBW values between the control group and the individual compound-treated groups. The compound is considered active at the specific dosage administered if the difference in plasma levels has *p* < 0.05; all compounds with *p* > 0.05 were reported as inactive.

### 2.3. Molecular docking studies

The crystallographic data for PPARγ co-crystallized with tesaglitazar were downloaded from the PDB data bank (http://www.rcsb.org/-PDB code: 1I7I). The studied compounds were built using the atomic partial charges calculated with the Gasteiger-Hückel method and their geometry was optimised by the Powell method available in the Maximin2 procedure to a gradient of 0.001 Kcal/mol.Å using the Tripos force field^21^. UCSF Chimaera 1.12^22^ (https://www.cgl.ucsf.edu/chimaera), a visualisation tool for molecular modelling and structural biology, was used to remove molecules, ions, and water and to minimise the structure of proteins and ligands, using the Gasteiger charges with 1000 steps of minimisation. Both ligands and receptor were then converted to pdbqt format. AutoDock Vina^23^ (http://vina.scripps.edu), an open-source programme for molecular docking, was used to investigate ligand-protein affinity. A grid box was centred (*x* = 17.949, *y* = 19.682, and *z* = 15.341) on the active site of PPARγ LBD, to cover the pocket with the main residues of receptor binding site. The best conformation was chosen with the lowest docked energy, based on complete docking search (ten runs). The interactions of PPARγ LBD with the ligands, hydrogen bonds, bond lengths, Root Mean Square Difference (RMSD) were analysed using Chimaera software. The docking solutions of tesaglitazar into binding site using AutoDock Vina converged consistently towards a unique binding mode within a 1.56 Å RMSD in comparison with the co-crystallized tesaglitazar and showed binding energy of −8.2 kcal/mol for protein–ligand complex.

## 3. Results and discussion

### 3.1. Chemistry

**Scheme 1.**
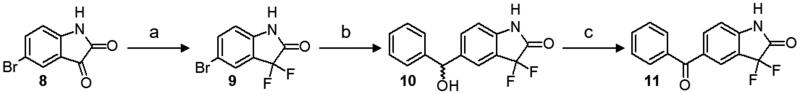
Synthesis of 5-benzoyl-3,3-difluoro-1,3-dihydro-2H-indol-2-one **11**. Reagents and conditions: (a) DAST, DCM, r.t.; (b) i: i-PrMgCl, n-BuLi, dry THF, 0 °C; ii: benzaldehyde, −78 °C; (c) Dess-Martin periodinane, DCM, r.t.

5-benzoyl-benzothiazol-2-one (**1**)^24^ and 6-benzoyl-benzothiazol-2-one (**2**)^25^, 6-benzoyl-benzothiazin-3-one (**3**) and 7-benzoyl-benzothiazin-3-one (**4**)^26^, 6-benzoyl-benzoxazin-3-one (**5**)^27^, and 7-benzoyl-benzoxazin-3-one (**6**)^28^ and finally 5-benzoyl-1,3-dihydro-2*H*-indol-2-one (**7**)^29^ were synthesised according to well-described procedures. 5-Bromoisatin (**8**) was easily converted to 5-bromo-3,3-difluoro-1,3-dihydro-2*H*-indol-2-one (**9**) using diethylaminosulfur trifluoride (DAST) in DCM^30^. Bromo derivative **9** was then subjected to a halogen–magnesium exchange reaction with in-situ produced isopropyl magnesium di-*n*-butyl lithium ate complex (*i*PrMgnBu_2_Li) at −10 °C. Benzaldehyde was then added at −78 °C to provide the corresponding benzyl alcohol **10** as a racemic mixture^31^. Finally, 5-benzoyl-3,3-difluoro-1,3-dihydro-2*H*-indol-2-one (**11**) was obtained by oxidation of alcohol **10** with Dess-Martin periodinane in DCM (Scheme 1)^32^.

**Scheme 2.**
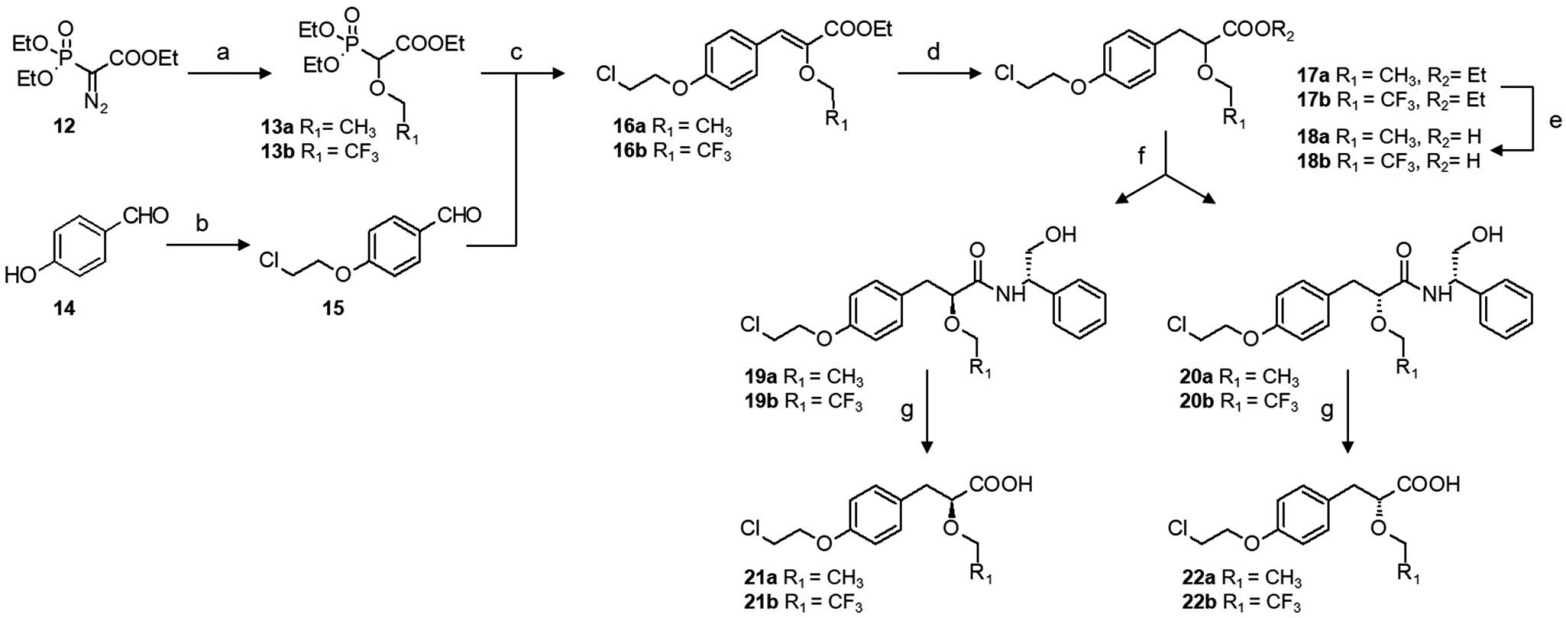
Synthesis of optically pure α–alkoxyphenylpropionic acids **21**, **22**. Reagents and conditions: (a) ethanol or trifluoroethanol [Rh(OAc)_2_]_2_,toluene, reflux; (b) 1,2-bromo-chloroethane, K_2_CO_3_, CH_3_CN, reflux; (c) NaH, dry THF, 0 °C to r.t.; (d) H_2_, Pd/C, ethanol, r.t.; (e) LiOH, THF/H_2_O, r.t.; (f) 1-ethyl-3–(3-dimethylaminopropyl)-carbodiimide hydrochloride, triethylamine, HOBT, (*S*)-(-)-2-phenylglycinol, DCM, 0 °C to r.t.; (g) 5 M H_2_SO_4_, dioxane/H_2_O, reflux.

**Scheme 3.**
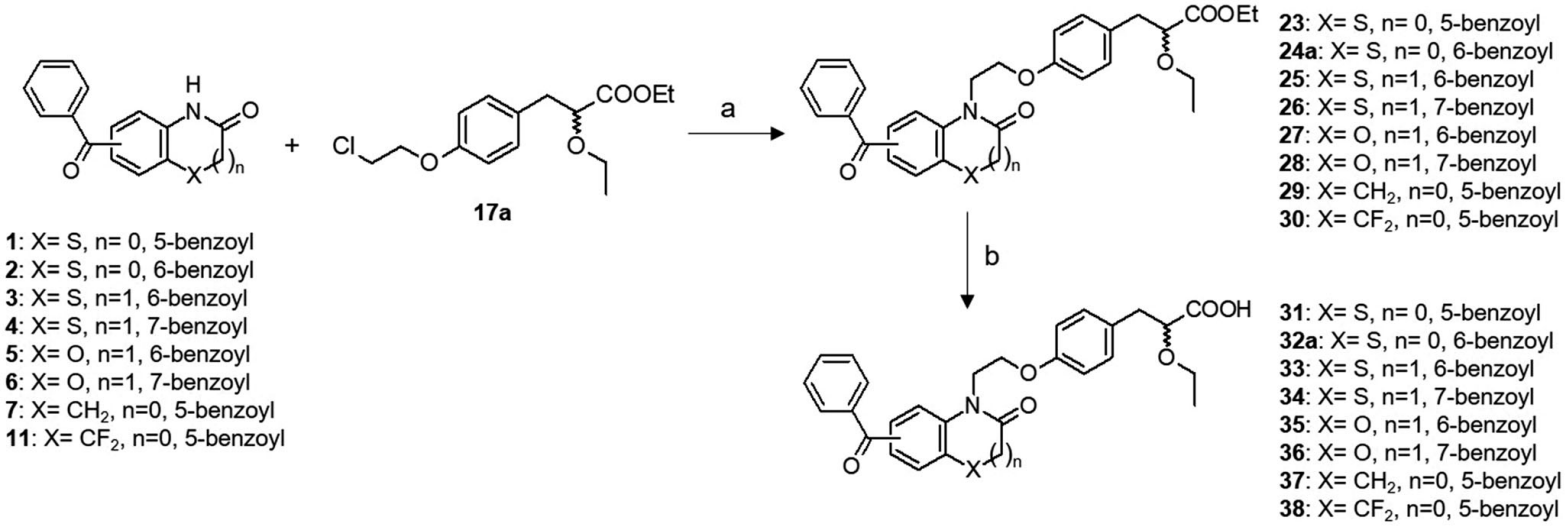
Synthesis of PPAR agonists **31–38**. Reagents and conditions: (a) K_2_CO_3_, DMF, 120 °C; (b) LiOH, THF/H_2_O, r.t.

**Scheme 4.**
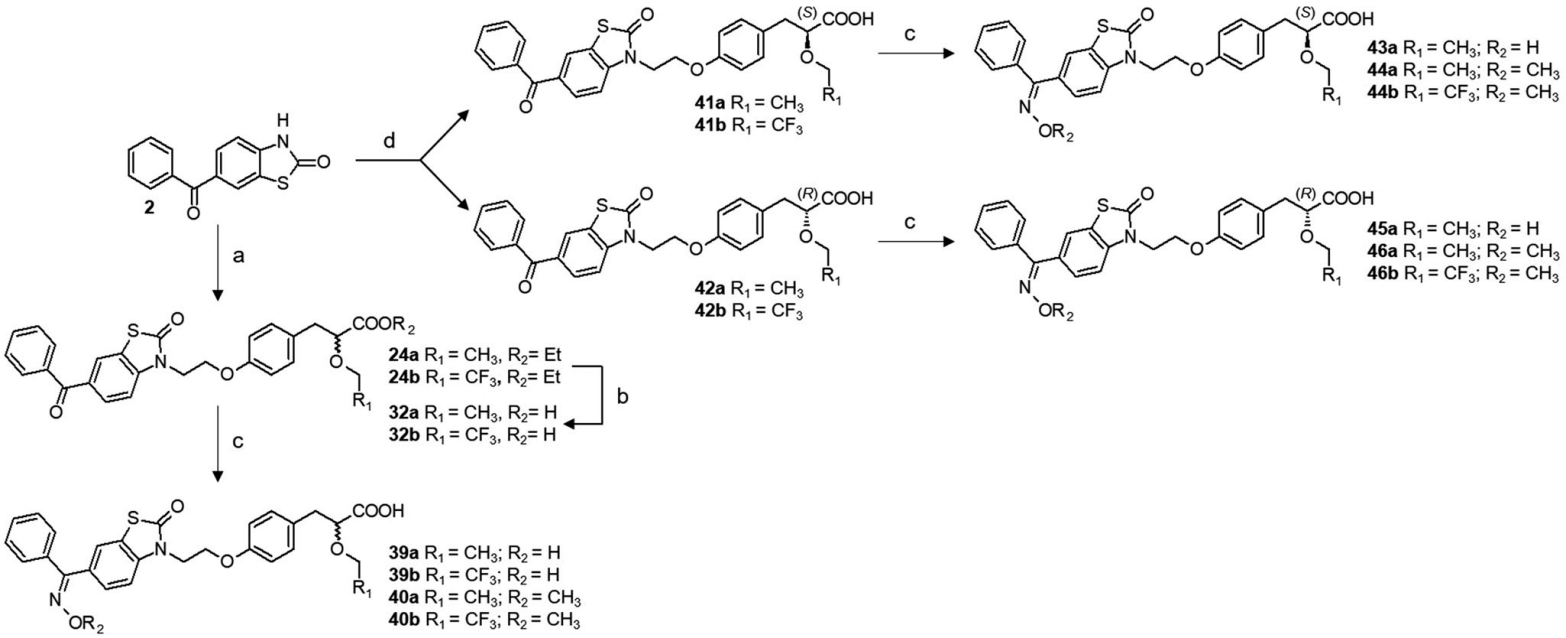
Synthesis of optically pure and oxime derived PPAR agonists **39–46**. Reagents and conditions: (a) K_2_CO_3_, **17a** or **17 b**, DMF, 120 °C; (b) LiOH, THF/H_2_O, r.t; (c) hydroxylamine hydrochloride or O-methylhydroxylamine hydrochloride, pyridine, reflux; (d) NaH, **21a,b** or **22a,b**, HMPA, r.t.

We previously described the synthesis of the key α-alkoxy propionic acid ester racemates **17a,b**^33^ and the resolution of the optically pure enantiomers **21a** and **22a**^15^. Herein, we also performed synthesis of the optically pure trifluoroethoxy derivatives **21 b** and **22 b** by chromatographic separation of diastereoisomeric amides **19 b** and **20 b** resulting of the reaction of the acid **18 b** with chiral (*S*)-2-phenylglycinol (Scheme 2). The condensation reaction between benzoyl heterocycles and racemic α-ethoxy propionic acid esters **17a,b** was classically carried out in the presence of potassium carbonate in DMF at 90 °C, and the desired acids **31–38** were obtained after lithium hydroxide mediated saponification (Scheme 3). Synthesis of enantiomerically pure **41a,b** and **42a,b** is outlined in Scheme 4. Condensation of **2** with the optically pure carboxylic acids **21a,b** and **22a,b** was performed under mild conditions, employing NaH in HMPA at room temperature, in order to prevent partial racemisation. Finally, racemic or optically pure aryl ketones **32**, **41** and **42** were further functionalised into the corresponding oxime derivatives **39**, **40** and **43–46** by treatment with hydroxylamine hydrochloride or O-methylhydroxylamine hydrochloride in pyridine. Surprisingly, while introduction of oxime and oxime ethers provided exclusively the *E* isomers (compounds **39a**, **40a,b**, **43a**, **44a,b**, **45a**, **46a,b**), assigned by NOE experiments, condensation of hydroxylamine hydrochloride with the trifluoroethyl derivative **32b** led to the formation of a racemic mixture of *E* and *Z* isomers **39b**.

### 3.2. In vitro *evaluation: binding studies and transactivation assays*

Compounds **31–38** were characterised by determining the binding affinity to human PPARγ using a competitive binding assay with [^3^H]rosiglitazone, the appropriate radioligand for PPARγ. The binding profiles of compounds were compared to the profiles of several references: rosiglitazone, a full PPARγ agonist and muraglitazar, a dual PPARα/γ agonist which was discontinued in clinical trials for greater incidence of oedema, heart failure, renal dysfunction and weight gain (Table 1). Except 6-benzoyl-benzothiazinone derivative **33**, all the tested compounds displayed potent binding affinities to PPARγ with *K_i_* values lower than 10 nM. The results are in agreement with the structural frameworks of compounds **31–38** and reference compounds which, like most PPAR activators, are conformed to a three-module structure, with a binder acidic group, an ethoxyphenyl linker and an effector end bearing a large hydrophobic tail part^1^. Molecular mechanisms will be addressed in the molecular docking studies in order to understand the large shift of binding affinity induced by the replacement of an oxygen atom by a sulphur atom in the 6-benzoyl substituted benzazinone series of compounds while no loss of affinity occurred with the 7-benzoyl derivatives.

**Table 1.** *In vitro* profile of compounds **31–38** on PPARγ affinity and PPARα/γ transactivation.


Cmpd	X	n	Pos^a^	*K*_i_ (nM)^b^	hPPAR gene reporter			
					αGal4 (%)^c^	αEC_50_ (nM)^d^	γGal4 (%)^c^	γEC_50_ (nM)^d^
**31**	S	0	5	8	81	256	145	61
**32a**	S	0	6	4	100	2400	89	15
**33**	S	1	6	220	19	10000	69	219
**34**	S	1	7	4	116	2050	97	37
**35**	O	1	6	3	97	10,000	119	2
**36**	O	1	7	1	80	10,000	113	80
**37**	CH_2_	0	5	6.5	100	10,000	60	40
**38**	CF_2_	0	5	4	89	10,000	75	5
**rosi^e^**				8	28	10,000	100	4
**mura^f^**				2	84	595	128	3
**WY14643**				/	100	10,000	N.A.	N.A.

^a^ Pos: position of the benzoyl substitution on the heterocycle.

^b^ hPPARγ binding affinity: *K*_i_ values were calculated according to the equation *K*_i_ = IC_50_/(1 + [L]/*K*_d_), where IC_50_ is the concentration of test compound required to inhibit 50% of the specific binding of the radioligand, [L] is the concentration of the radioligand used, and *K*_d_ is the dissociation constant for the radioligand at the PPARγ receptor.

^c^ Efficiency: Emax was the maximal PPAR fold activation relative to maximum activation obtained with WY14643 (10 µM) and rosiglitazone (1 μM) corresponded to 100% in GAL4 chimeric hPPARα and hPPARγ system.

^d^ EC_50_^:^ Concentration required to induce 50% maximum activity of tested compound.

^e^ Rosiglitazone.

^f^ Muraglitazar.

NA: not active.

Functional activity of compounds **31–38** was measured in a transient transfection assay using human pGAL4hPPARα and human pGAL4hPPARγ. The results are given in Table 1. All the target compounds were tested as racemic mixtures and were functionally inactive at 10 µM against human PPARδ. The functional profiles of test compounds were compared to the same references as for binding studies for PPARγ and to WY14,643, a full fibrate PPARα agonist. Benzothiazol-2-one derivatives **31** and **32a** were considered as dual full PPARα/γ agonists with potency ≥ 80%. 5-Benzoyl derivative **31** exhibited better α functional activity than its 6-benzoyl counterpart **32a** and proved to interact better with the PPARα ligand binding domain (LBD) (compounds **31** and **32a** have respective PPARα EC_50_ of 256 and 2400 nM). In the benzoxazin-3-one series, 6- and 7-benzoyl substituted derivatives **35** and **36** were equipotent with a very potent full PPARγ agonist pharmacologic profile (potency > 100%) and a full agonist profile at 10 µM on the PPARα receptor subtype equivalent to the reference WY14,643. Benzothiazin-3-one analogues displayed more contrasted results with functional activity ranging from 37 nM to 219 nM on PPARγ and from 2 µM to 10 µM on PPARα. While 7-benzoyl derivative **34** acted as dual full PPARα/γ agonist, its 6-benzoyl counterpart **33** should be considered as partial PPARγ agon since it displayed lower potency (≤ 70%) on PPARγ and was found totally inactive (potency ≤ 20%) against PPARα. Functional activity results obtained for compound **33** were in accordance with the binding studies. The oxygen atom replacement by a sulphur atom in the 6-benzoyl substituted series of compounds did not seem to be well tolerated for PPARα/γ activity in comparison with the steric bulk of 7-benzoyl substituted derivatives. In order to assess the impact of oxygen or sulphur contained heterocycles on PPAR functional activity, 5-benzoyl-1,3-dihydro-2H-indol-2-one **37** and its 3,3-difluoro derivative **38** were designed and synthesised. Replacement of a C–H bond with fluorine may lead to special beneficial effects which are linked with its small size, its high electronegativity and low polarizability and its lipophilicity^34^. These structural modifications led to a slight decrease in γ functional efficiency (≤ 75%) while maintaining good PPARγ potency (EC_50_ ≤ 40 nM), suggesting that compounds **37** and **38** could be considered as partial PPARγ agonists. However, direct comparison between compounds **37**, **38** and **32a** revealed that replacement of the sulphur atom by a methylene derivative weakens the ligand–PPARα interaction and likely decreases activity. We decided to select and develop for further studies, compound **32a** bearing a benzothiazol-2-one which seems to be the most tolerant of the tested heterocycles and for which acylation in position 6 was chemically easier than its 5-benzoyl counterpart.

Resolution of the racemate **32a** into its optical antipodes first led to compound **41a**, which corresponded to the (*S*)-enantiomer and possessed very potent binding affinity to PPARγ with K_i_ value lower than 1 nM and acted as full dual PPARα/γ agonist (Table 2). In contrast, its (*R*)-configured counterpart **42a** proved to interact more weakly with the PPARγ LBD with K_i_ value of 70 nM and was found to be a selective partial PPARγ agonist with no activity for the PPARα subtype. These data were in accordance with previous studies describing PPAR agonists containing (*S*)-ethoxypropanoic acid which displayed higher activities than the (*R*)-stereoisomer analogues at both PPARα and PPARγ receptor subtypes^35^.

**Table 2.** *In vitro* profile of compounds **32, 39–46** on PPARγ affinity and PPARα/γ transactivation.


Cmpd	X	R	Stereo	*K*_i_ (nM)^b^	hPPAR gene reporter			
					αGal4 (%)^c^	αEC_50_ (nM)^d^	γGal4 (%)^c^	γEC_50_ (nM)^d^
**32a**	O	CH_3_	*Rac*	4	100	2400	89	15
**39a**	NOH	CH_3_	*Rac*	2	129	10,000	91	24
**39b**	NOH	CF_3_	*Rac*	165	29	10,000	44	10
**40a**	NOCH_3_	CH_3_	*Rac*	4	159	129	87	2
**40b**	NOCH_3_	CF_3_	*Rac*	1.3	56	180	95	7
**41a**	O	CH_3_	*S*	0.5	166	522	142	8
**42a**	O	CH_3_	*R*	70	0	10,000	39	300
**43a**	NOH	CH_3_	*S*	0.5	153	10,000	123	16
**44a**	NOCH_3_	CH_3_	*S*	0.7	154	32	115	2
**44b**	NOCH_3_	CF_3_	*S*	3	59	525	150	0.9
**45a**	NOH	CH_3_	*R*	210	30	10,000	66	799
**46a**	NOCH_3_	CH_3_	*R*	86	124	1480	87	558
**46b**	NOCH_3_	CF_3_	*R*	33	117	1665	116	9
**rosi^e^**				8	28	10,000	100	4
**mura^f^**				2	84	595	128	3
**WY14643**				/	100	10,000	N.A.	N.A.

^a^ Pos: position of the benzoyl substitution on the heterocycle.

^b^ *K*_i_ values were calculated according to the equation *K*_i_ = IC_50_/(1 + [L]/*K*_d_), where IC_50_ is the concentration of test compound required to inhibit 50% of the specific binding of the radioligand, [L] is the concentration of the radioligand used, and *K*_d_ is the dissociation constant for the radioligand at the PPARγ receptor.

^c^ Potency: Emax was the maximal PPAR fold activation relative to maximum activation obtained with WY14643 (10 µM) and rosiglitazone (1 μM) corresponded to 100% in GAL4 chimeric hPPARα and hPPARγ system.

^d^ EC_50_^:^ Concentration required to induce 50% maximum activity of tested compound.

^e^ Rosiglitazone.

^f^ Muraglitazar.

NA: not active.

Functionalization of the ketone tail portion of the acyl group with O-methyl oxime ether or introduction of a trifluoroethoxy group in previous PPAR agonist series developed in our laboratory were found to respectively enhance PPARα and decrease PPARγ functional activity^15^^,^^33^^,^^36^. These chemical modulations were then applied by oxime forming reactions on the benzoyl part of ethoxy **32a** or trifluoroethoxy **32 b** racemates in order to assess the impact of the nature of the substituents on PPAR transactivation (Table 2). As previously reported, incorporation of O-methyl oxime ether led to compounds **40a,b** exhibiting better α functional activity than the carbonyl counterpart **32a** and especially the non-substituted oximes **39a,b**. Introduction of trifluoroethoxy group led to compound **39 b** displaying a selective partial PPARγ agonist profile and to compound **40b** acting as dual PPARα/γ agonist with partial PPARα and full PPARγ agonist activities. These results confirm that the incorporation of substituents such as methyl oxime ether and trifluoroethoxy group strengthens the ligand–PPARα interaction and likely increases activity while maintaining good PPARγ potency. We have next evaluated optically pure stereoisomers of racemates **39a** and **40a,b** for both binding and transactivation studies. As previously reported, (*S*)-configured derivatives **43a**, **44a,b** proved to interact better with the PPARγ LBD with binding affinities 10- to 400-fold more potent than their (*R*)-counterparts **45a**, **46a,b**. They are also highly potent agonists of both human PPARα and γ in cellular assays except for non-substituted oxime derivatives **43a** and **45a** which can be considered as selective PPARγ agonists.

In the (*S*)-series of stereoisomers, introduction of an O-methyl oxime ether considerably improved PPARα efficiency and potency while replacement of ethoxy group by its trifluoro analogue led to compound **44 b** with lower functional activity, characterised by a partial PPARα agonist profile. These chemical modulations didn’t affect the PPARγ receptor subtype leading to compounds with very potent PPARγ agonist profile.

### 3.3. Molecular docking studies

**Figure 2.**
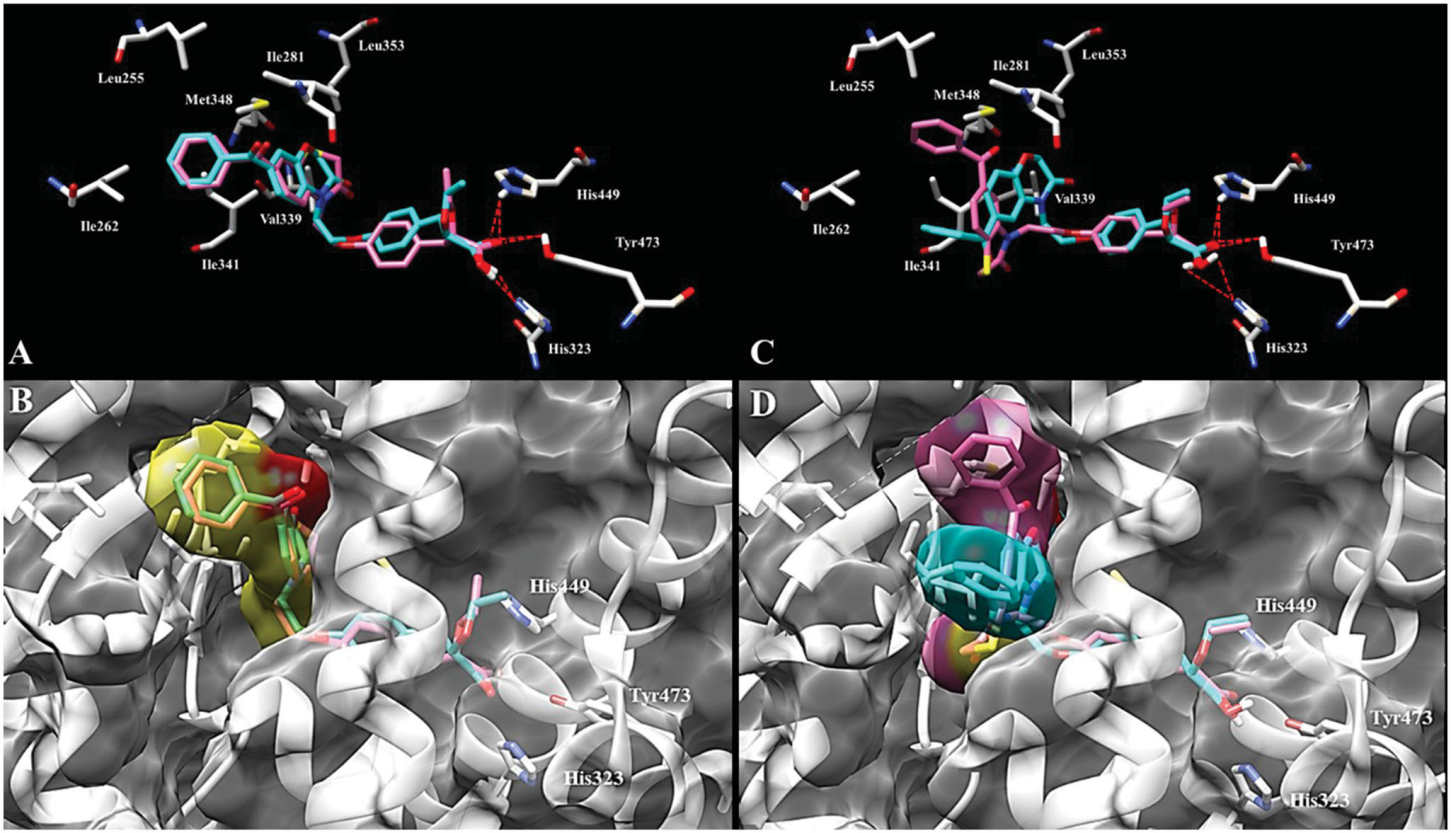
Predicted binding mode of (A) **(*S*)-34** (pink) and **(*S*)-36** (cyan) and (C) (***S*)-33** (pink) and **(*S*)-35** (cyan) enantiomers with PPARγ LBD (PDB id 1I7I). Key amino acid side chains are shown in white stick format and are labelled and hydrogen bonds are shown in red dotted lines. Overlapping and molecular surface maps of (B) **(*S*)-34** (pink) and **(*S*)-36** (cyan) and (D) (***S*)-33** (pink) and **(*S*)-35** (cyan) enantiomers with PPARγ LBD (PDB id 1I7I). The protein and key amino acid side chains are shown in white rounded ribbon and white stick format, respectively. The molecular surface maps are shown in grey.

Molecular modelling studies were conducted to understand the large shift of binding affinity induced by the replacement of an oxygen atom by a sulphur atom in the 6-benzoyl substituted benzazinone series of compounds while no loss of affinity occurred with the 7-benzoyl derivatives. Whereas compounds **33–36** were synthesised and evaluated as racemic mixtures, only (*S*)-enantiomers have been analysed in docking studies, since (*S*)-stereoisomers displayed the best pharmacological profiles described in the literature. Crystal structure of PPARγ (PDB id: 1I7I)^37^ co-crystallized with tesaglitazar was downloaded from PDB database. The binding poses of 7-benzoyl benzazinones **34** and **36** with the LBDs of PPARγ are shown in Figure 2(A). In addition to the classical hydrogen bondings with His323, His449 and Tyr473, it was apparent that the benzoyl moiety and the benzoxazin-3-one or benzothiazin-3-one cores are buried in a binding cavity of PPARγ LBD composed of a cluster of hydrophobic residues (Leu255, Ile262, Ile281, Val339, Ile341, Met348 and Leu353). In the 7-benzoyl series of compounds, each sulphur **34** or oxygen **36** derivative is well superimposed with each other in the PPARγ LBD with the heterocyclic cores deeply oriented in the hydrophobic pocket and the benzoyl moieties directed towards the solvent (Figure 2(B)). In the 6-benzoyl series of compounds, benzoxazin-3-one **35** performed the same type of interactions as its 7-benzoyl counterpart with the heterocycle core in the hydrophobic cavity and the benzoyl exposed to the solvent (Figure 2(C,D), cyan molecule). However, the benzothiazin-3-one derivative **33** didn’t hinder at all the hydrophobic pocket since the set of binding poses adjusts both the phenyl part of benzoyl and the benzothiazinone sulphur atom towards the solvent (Figure 2(C,D), pink molecule). The unexpected PPARγ binding affinity of compound **33** can be explained in terms of deformed torsional angles which are more pronounced in the six-membered rings containing sulphur atoms relative to those containing oxygen atoms.

### 3.4. In vivo *biological evaluation*

The *in vivo* effect of test compounds was determined by measuring glycaemia, triglyceridemia, insulinemia, and body weight using ob/ob mice (Table 3). The ob/ob mice were obese, insulin resistant with hypertriglyceridaemia and hyperglycaemia, and have been used as a rodent model of obesity-induced insulin resistance. In this model, mice were dosed orally with either synthesised compounds or standard reference compounds (rosiglitazone and muraglitazar) at 8.4 µMkg^−1^ and 3 mgkg^−1^ respectively for 4 days. As a side effect index, body weight was also measured daily, and body weight variation was calculated as a percentage of the difference in body weight between days 4 and 0. The results were normalised to those obtained with rosiglitazone in the same experiment, which was set to 100%. Benzoyl derivatives **32a**, **41a** and **42a** were not evaluated *in vivo* with regard to their low absorption values with CaCO_2_ cells (< 25%), an *in vitro* model predicting membrane permeability^38^. In this model, oxime derivatives displayed good percentages of absorption ranging from 65% to 100%, except compound **45a** with only 31% in CaCo_2_ permeability. Racemate **39a** bearing an oxime function displayed *in vivo* profile similar to reference compounds, but without promoting body weight gain compared with rosiglitazone and muraglitazar. Interestingly, incorporation of O-methyl oxime ether led to compound **40a** showing a slight improvement of the pharmacological activity in terms of reducing triglyceridemia, glycaemia and insulinemia. However, no improvement was obtained in terms of body weight gain showing the same level as muraglitazar and suggesting the same side effects. Introduction of a trifluoroethoxy group led to less potent racemates **39 b** and **40 b** but with a sharp decrease in body-weight gain compared with rosiglitazone. These results are consistent with the results obtained in the PPARα and γ gene reporter assays. Indeed, incorporation of substituents such as methyl oxime ether and trifluoroethoxy group are benefiting for PPAR activity regulation and so for effective management of dyslipidemia without inducing body weight gain. The same results were observed with the (*S*)-derivative **44 b** in which O-methyl oxime ether allowed significant decreases in serum triglyceride, glucose and insulin levels compared with its oxime counterpart **43a**. The CF_3_ group incorporation, beyond keeping a good *in vivo* efficacy, provided a robust reduction of body weight induced with compound **44a**, strengthening the beneficial pharmacological effect of C-H bond replacement with fluorine. Compound **44 b** may be considered as a SPPARγM since this full PPARγ agonist retains the insulin-sensitizing and glucose-lowering actions while mitigating or eliminating the undesirable side effects. Finally, only the (*R*)-stereoisomer **45a** was engaged in the *in vivo* experiments and was found inactive as the whole of PPAR ligands described in the literature^1^.

**Table 3.** *In vivo* efficacy of compounds **39a,b, 40a,b, 43a, 44a,b, 45a** in *ob/ob* mice animal models.

		Change	(%)^a^	
Cmpd	TG^b^	Gly^c^	Ins^d^	ΔBW^e^
**39a**	−69	−49	−34	−10
**39b**	−30	−18	10	75
**40a**	−83	−58	−76	188
**40b**	−54	−37	−55	−70
**43a**	−33	−55	−29	113
**44a**	−79	−46	−81	177
**44b**	−69	−36	−89	−17
**45a**	21	−7	51	328
**rosi^f^**	−46	−57	−73	100
**mura^g^**	−34	−46	−46	206

^a^Percent change versus control for ob/ob mice at day 4.

^b^Triglycerides.

^c^Glycemia.

^d^Insulin.

^e^Body weight variation between day 0 and day 4 are expressed in% of rosiglitazone in the same study.

^f^Rosiglitazone.

^g^Muraglitazar.

## 4. Conclusion

Dual PPARα/γ agonists that were developed to target hyperlipidaemia and hyperglycaemia in type 2 diabetes patients, caused cardiac dysfunction or other adverse effects as fluid retention and body-weight gain. Thus, there was significant interest in the design of novel partial agonists and/or selective PPAR modulators (SPPARMs) corresponding to a safer generation of PPAR ligands that evoke fewer side effects while preserving insulin-sensitizing potential. In this context we have reported the design of various nitrogen heterocycles containing α-ethoxyphenylpropionic acid chain **31–38** as PPAR agonists. Except 6-benzoyl-benzothiazinone derivative **33**, all the tested compounds displayed potent binding affinities to PPARγ with K_i_ values lower than 10 nM. Molecular docking studies showed that the benzothiazin-3-one heterocycle wasn’t able to correctly accommodate its 6-benzoyl substituent in the hydrophobic pocket. 6-Benzoyl-benzothiazol-2-one which was the most tolerant of the tested heterocycles was further investigated to identify new PPAR ligands with the best *in vitro* pharmacological profiles. Incorporation of O-methyl oxime ether and trifluoroethoxy group followed by enantiomeric resolution led to the (*S*)-stereoisomer **44 b**. On the gene reporter assays, this compound displayed a very potent full PPARγ agonist pharmacologic profile and a weak partial agonist profile on the PPARα receptor subtype. This kind of pharmacological profile, common to numerous PPARγ ligands, suggested a potent insulin sensitivity and robust glucose-lowering activity but certainly shadowed by the risk for fluid retention and weight gain. However, after 4 days of oral treatment with **44 b**, ob/ob mice did not gain weight compared with rosiglitazone as reference, while keeping high efficacy on the decreasing blood parameters. Compound **44 b** seems to differentially induce specific PPAR receptor effects depending on the cellular context with high anti-diabetic properties and low potency in terms of effects on adipose generation, fluid retention and weight-gain as characterised by SPPARMs. Complementary studies will be performed to evaluate the effect of compound **44 b** in term of osteoporosis and cardiac function to reinforce its safety profile. In conclusion, 6-benzoyl-benzothiazol-2-one is a scaffold that could be substituted to develop therapeutic agents for diabetes and/or serve as a lead compound for further drug design studies targeting PPAR-γ and PPARα for effective management of type 2 diabetes and dyslipidemia without inducing weight gain.
